# Predicting the response of triple negative breast cancer to neoadjuvant systemic therapy *via* biology-based modeling and habitat analysis

**DOI:** 10.1038/s41598-025-25989-z

**Published:** 2025-11-26

**Authors:** Casey E. Stowers, Chengyue Wu, Clinton Yam, Jingfei Ma, Gaiane M. Rauch, Thomas E. Yankeelov

**Affiliations:** 1 Oden Institute for Computational Engineering and Sciences, The University of Texas at Austin, Austin, TX USA; 2 Livestrong Cancer Institutes , The University of Texas at Austin , Austin, TX USA; 3 Departments of Imaging Physics , The University of Texas MD Anderson Cancer Center , Houston, TX USA; 4 Departments of Abdominal Imaging , The University of Texas MD Anderson Cancer Center , Houston, TX USA; 5 Department of Breast Imaging , The University of Texas MD Anderson Cancer Center , Houston, TX USA; 6 Departments of Biomedical Engineering , The University of Texas at Austin , Austin, TX USA; 7 Departments of Diagnostic Medicine , The University of Texas at Austin , Austin, TX USA; 8 Departments of Breast Medical Oncology , The University of Texas MD Anderson Cancer Center, Houston, TX USA; 9 Departments of Biostatistics, The University of Texas MD Anderson Cancer Center, Houston, TX USA; 10 Institute for Data Science in Oncology , The University of Texas MD Anderson Cancer Center , Houston, USA TX

**Keywords:** Breast cancer, Computational science, Computational models, Chemotherapy, Magnetic resonance imaging, Cancer

## Abstract

Despite being the standard-of-care treatment, neoadjuvant therapy (NAT) attains a complete response only in approximately half of the patients with triple negative breast cancer. Thus, methods to predict and optimize patient response to NAT are needed. Previously, we employed patient-specific MRI data to calibrate a biology-based mathematical model that describes cell movement, proliferation, and death due to drug at the tumor level and cell proliferation at an image voxel level. We now extend our approach by using MRI data to group voxels into “habitats” whereby tumor cells of a habitat share the same proliferation. With this approach, we now calibrate habitat-informed proliferation rates for each habitat rather than local proliferation rates. When comparing error in tumor cell number and volume at the time of calibration, the local calibration has significantly (*p* < 0.05) lower error than the habitat-informed calibration. However, the habitat-informed predictions of a future timepoint have significantly lower error than the local predictions. Compared to the local calibration, the habitat-informed calibration also requires fewer parameters, reducing the calibration time by a factor of 17. These results suggest that a habitat-informed calibration can provide both accurate and efficient predictions of breast cancer response to NAT.

**Supplementary Information:**

The online version contains supplementary material available at 10.1038/s41598-025-25989-z.

## Introduction

Neoadjuvant therapy (NAT) is the standard of care for treating patients with locally advanced triple-negative breast cancer (TNBC)^1,2^. Unfortunately, only about half of the patients achieve a pathological complete response (pCR, defined as absence of any residual cancer cells by pathological examination of the resected tissue and sampled lymph nodes^3^ by the completion of NAT^4^. The addition of immunotherapy can boost the patient pCR rate to about two thirds, but at the cost of increased toxicity^5^. As patients with a pCR to NAT are less likely to experience disease recurrence and death^1,2^, it is imperative to develop methods that can predict and optimize patient response to NAT in an efficient, accurate, and interpretable fashion.

We have previously shown that a biology-based model built on a reaction-diffusion equation can accurately capture patient-specific tumor growth and response to NAT^6,7^. The model includes a diffusion term describing cell movement (coupled to tissue mechanics) and a reaction term describing tumor cell proliferation (*via* logistic growth) and a term for response to therapy (*via* exponential death coupled to drug concentration). As the terms in these models describe specific biological behavior, the model predictions are readily interpretable. This approach has achieved high accuracy for predicting the response of locally advanced breast cancer to NAT^8,9^, and shown potential for optimizing therapeutic schedules^10–12^. The parameters in the diffusion, proliferation, and therapy response terms can be calibrated on a patient-specific basis using magnetic resonance imaging (MRI) data acquired before and during NAT. However, the parameters in these models are often calibrated either globally (i.e., one value per tumor)^13–15^ or locally (i.e., one value per tumor voxel)^8,9,16–18^. Global parameters are limited in their spatial accuracy and ability to capture tumor heterogeneity, while local parameter calibration can be computationally expensive and may require reduced order modeling methods to increase clinical feasibility^19^. Here, we seek a tradeoff between global and local parameter calibration by segmenting tumors into regions with constant model parameters.

To identify subregions of interest in the tumors, we employ habitat analysis, a method in which MR images have been used to identify “habitats” with distinct characteristics within tumors^20,21^. To identify habitats, clustering algorithms (e.g., *k*-means) are applied to multiparametric MRI data across a patient cohort^20,21^. Habitats can be linked to biological meaning *via* the images used to generate them. In the pre-clinical setting, the physical meaning assigned to the habitats based on the imaging characteristics can be confirmed using histology^22–24^. In the breast cancer clinical setting, habitats have been used to predict pCR status^25,26^, recurrence free survival^27^, disease free survival^28^, therapy response^29^, BRCA1 mutation status^30^, and distinguish TNBC from non TNBC tumors^31^. While habitats are often analyzed to directly predict metrics such as pCR status or therapy response, they do not inherently elucidate the mechanisms that drive particular tumor behaviors and cannot be used for therapy optimization due to this lack of interpretability^25,26,29^. More recently, preliminary efforts have employed habitats to initialize biology-based mathematical models to predict tumor dynamics^32,33^.

In this contribution, we used quantitative diffusion weighted and dynamic contrast enhanced MRI data to identify regions with similar features across the imaging timecourse and assign them as distinct habitats. Each of these habitats will be calibrated and assigned a unique proliferation rate. By calibrating the biology-based model parameters on a habitat basis, we dramatically reduced the number of parameters that need to be calibrated compared to a voxel-based approach, thus with a high efficiency for predictions of tumor response to therapy.

## Methods

### Patient treatment and imaging timeline

Patient data (*n* = 138) for this study was obtained from the Institutional Review Board-approved prospective clinical trial “A Robust TNBC Evaluation FraMework to Improve Survival” (ARTEMIS; ClinicalTrials.gov, NCT02276443, first date posted 28/10/2014) at MD Anderson Cancer Center. We confirm that informed consent was obtained from all patients and/or their legal guardian(s) between April 2018 and May 2021 and that all methods were carried out in accordance with all guidelines and regulations outlined in the IRB-approval. All patients received four cycles of Adriamycin/Cytoxan (A/C) with multiparametric MRI scans before (V1), after two cycles (V2), and after four cycles (V3) of A/C. At the time of V3, tumor volume was determined *via* ultrasound imaging. If a patient had at least a 70% reduction in tumor volume, they then received 12 weekly cycles of Taxol. Patients with less than 70% reduction in tumor volume received experimental therapies per the ARTEMIS trial guidelines. After completing the second course of therapy, patients received surgery and their pCR status was determined. In this study, we only analyzed the pCR status of patients who received Taxol (*n* = 101) to maintain consistency in treatment received by patients included in the pCR analysis. We summarize patient age, second course of therapy, histologic type, T and N category, and pCR status in Supplemental Table S1.

### Patient imaging data

**Fig. 1:**
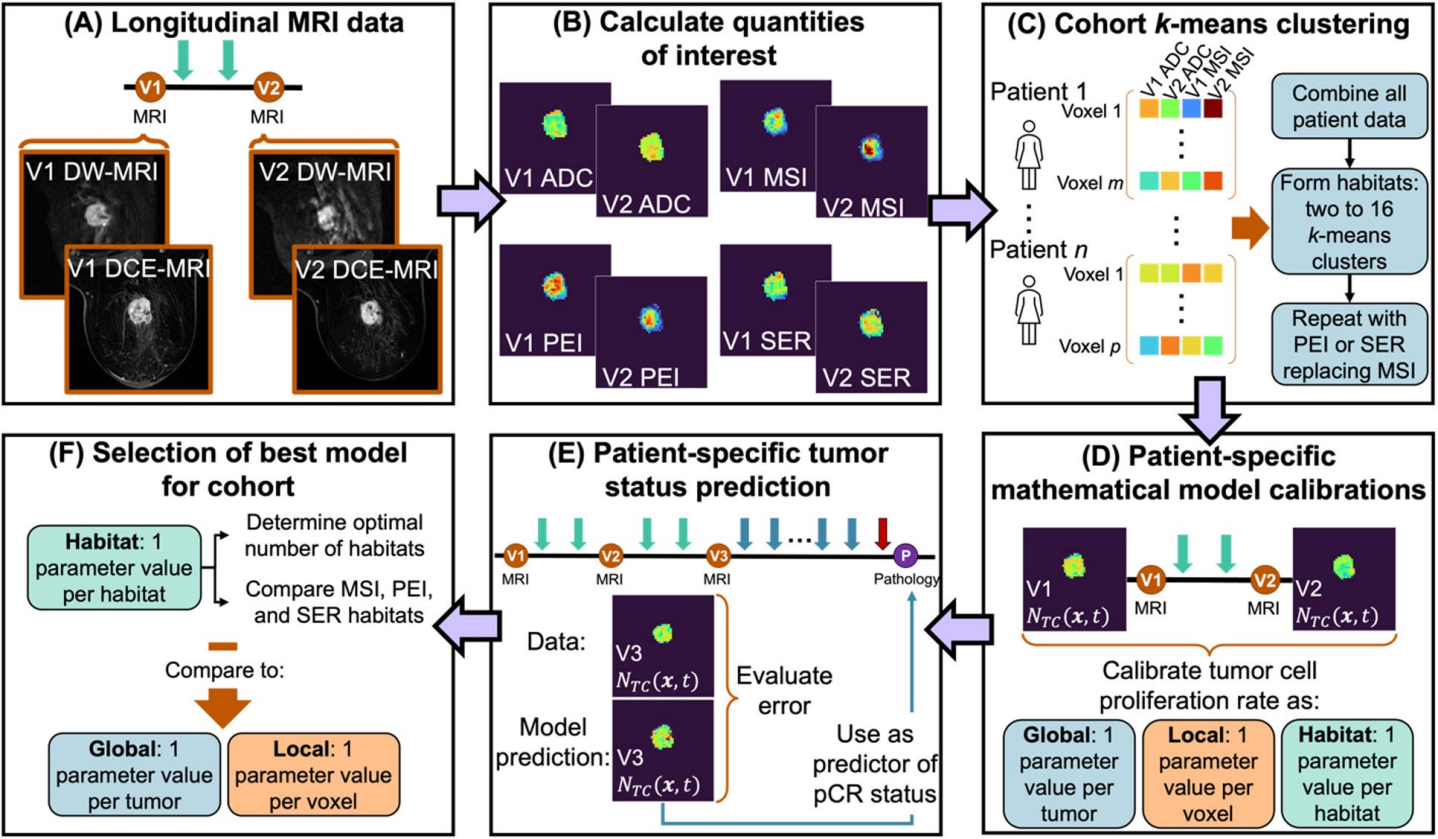
In Panel (**A**) we begin with the longitudinal MRI data acquisition which includes diffusion weighted (DW-) and dynamic contrast enhanced (DCE-) MRI. Visit 1 (V1) refers to the pre-NAT time point, while visit 2 (V2) occurs after the patient receives two cycles of NAT. Then, in Panel (**B**) we calculate the apparent diffusion coefficient (ADC) at each visit from the DW-MRI data and the maximum slope of increase (MSI), positive enhancement integral (PEI), and signal enhancement ratio (SER) from the DCE-MRI data. The quantities of interest are then used in a cohort *k*-means clustering analysis to define the habitats in Panel (**C**) We form vectors of longitudinal ADC data and one of MSI, PEI, or SER for every tumor voxel in each patient. All voxels from all patients in the cohort are combined into a matrix, which is then standardized and used to form two to 16 *k*-means clusters for each of the three DCE-MRI quantities of interest. After forming habitats, we calibrate our biology-based mathematical model on a patient specific basis in Panel (**D**) We calibrate using the V1 and V2 MRI data to obtain patient specific cell proliferation and drug efficacy values. The cell proliferation values can be calibrated in three ways: (1) globally, giving one parameter value per tumor, (2) locally, giving one parameter value per voxel, or (3) by habitat, giving one parameter value per habitat. In Panel (**E**) we use the calibrated parameter values in the biology-based model to predict tumor status at visit 3 (V3), which occurs after two additional cycles of NAT. Error is determined by comparing the predictions to the V3 MRI data, and the ability of the V3 predictions to predict pathological complete response (pCR) at the conclusion of NAT. After evaluating the patient-specific model predictions, we select the optimal model calibration scheme across the cohort in Panel (**F**) We first consider the optimal number of habitats to select and the differences in errors between the DCE-MRI data types used to form the habitats. Second, we compare the optimal habitat number calibrations to the global and local calibrations.

The details of the MRI acquisition and image registration have been previously reported^8^. Briefly, each imaging visit (i.e., V1, V2, and V3) included diffusion weighted (DW-) MRI scans with two *b*-values (*b* = 100, 800 s/mm^2^) and a dynamic contrast-enhanced (DCE-) MRI series with a median (range) of 42 (36–50) frames acquired with a temporal resolution of 11.1 (7.6–12.4) seconds (see Fig. 1A). Tumors were segmented manually at each visit^8,34,35^by board-certified breast radiologists. We defined the tumor region of interest (ROI) for this study as the union of the V1 and V2 segmentations. To ensure the tumors are comparable in space and time, we applied the registration pipeline developed by Jarrett et al.^36^. Specifically, different images acquired at each visit were aligned using a rigid registration. For inter-registration, the V1 and V3 images were aligned to the V2 images *via* non-rigid registration with a rigidity penalty on the tumor. The penalty ensured that large changes were not made to the tumor shape or size when aligning between visits.

As seen in Fig. 1B, we next computed the apparent diffusion coefficient (ADC) map by fitting the DW-MRI data to Eq. (1):

1 $$\:{S}_{b}={S}_{0}{e}^{-b\cdot\:ADC},$$

where $\:{S}_{b}$ is the signal intensity at the given *b*-value and $\:{S}_{0}$ is the signal intensity for *b* = 0 s/mm^[236^. We then estimated tumor cell number maps, $\:{N}_{TC}(\varvec{x},t)$, at each visit through Eq. (2), which defines an inverse relationship between the ADC and tumor cell number^37^:

2 $$\:{N}_{TC}\left(\varvec{x},t\right)=\theta\:\left(\frac{AD{C}_{w}-ADC\left(\varvec{x},t\right)}{AD{C}_{w}-AD{C}_{min}}\right),$$

where $\:AD{C}_{w}$ is the ADC of water at 37 °C (3.0 × 10^− 3^ mm^2^/s)^38^, $\:AD{C}_{min}$ is the minimum ADC value in a patient’s tumor across all visits, and $\:\theta\:$ is the physical carrying capacity, calculated as the number of spherical cells of radius 10 μm that can fit in a voxel with a packing density of 0.7405^36,39^.

Three semi-quantitative maps were calculated from the DCE-MRI data. These maps were selected as they have been shown to correlate with response^40^ and are therefore useful measures to incorporate into a biology-based model. To ensure consistency across the cohort, we first resampled all patient DCE-MRI timecourses to 360 s with a temporal resolution of 10 s. We define $\:DCE(\varvec{x},\tau\:,t)$ as the DCE-MRI signal at position $\:\varvec{x}$, time in the DCE timecourse $\:\tau\:$, and image scanning time *t*. We also obtain the normalized DCE-MRI signal, $\:DC{E}_{N}(\varvec{x},\tau\:,t)$, *via* Eq. (3):

3 $$\:DCE_{N} (x,\tau \:,t)\: = \frac{{DCE\left( {x,\tau \:,t} \right) - \mathop {\min }\limits_{{\tau \:}} DCE(x,\tau \:,t)\:}}{{\mathop {\max }\limits_{{\tau \:}} DCE\left( {x,\tau \:,t} \right) - \mathop {\min }\limits_{{\tau \:}} DCE(x,\tau \:,t)\:}},t{\text{ }} = {\text{ }}V1,{\text{ }}V2,{\text{ }}V3$$

The normalized DCE-MRI signal was used to calculate the first quantity of interest, the maximum slope of increase (MSI), *via* Eq. (4):

4 $$\:MSI\left( {x,t} \right) = \mathop {\max }\limits_{{\tau \:}} \frac{{DCE_{N} \left( {x,\tau \: + \Delta \:\tau \:,t} \right) - DCE_{N} (x,\tau \:,t)}}{{\Delta \:\tau \:}},t{\text{ }} = {\text{ }}V1,{\text{ }}V2,{\text{ }}V3$$

where is the time between the two successive DCE frames (i.e., 10 sec). The second quantity of interest is the positive enhancement integral (PEI), which was calculated using Eq. (5):

5 $$\:\:PEI\left( {x,t} \right) = \Delta \:\tau \:\sum {\:_{{\tau \:}} } DCE_{N} \left( {x,\tau \:,t} \right),t{\text{ }} = {\text{ }}V1,{\text{ }}V2,{\text{ }}V3$$

The third and final quantity of interest is the signal enhancement ratio (SER), which is calculated using Eq. (6)^41^:

6 $$\:\:SER\left( {x,t} \right) = \frac{{S_{1} \left( {x,t} \right) - S_{0} \left( {x,t} \right)}}{{S_{2} \left( {x,t} \right) - S_{0} \left( {x,t} \right)}},t{\text{ }} = {\text{ }}V1,{\text{ }}V2,{\text{ }}V3$$

where $\:{S}_{0}\left(\varvec{x},t\right)$ is the average of the $\:DCE\left(\varvec{x},\tau\:,t\right)$ frames at 0, 10, 20 s (pre-contrast), $\:{S}_{1}\left(\varvec{x},t\right)$ is the average of the $\:DCE\left(\varvec{x},\tau\:,t\right)$ frames at $\:\tau\:=$110, 120, 130 s (early post contrast, during wash-in and peak enhancement), and $\:{S}_{2}\left(\varvec{x},t\right)$ is the average of the $\:DCE\left(\varvec{x},\tau\:,t\right)$ frames at $\:\tau\:=$ 340, 350, 360 s (late post contrast, during wash out).

### Longitudinal habitat analysis

Three methods of habitat analysis were investigated using ADC and each of the three DCE-MRI-derived quantities of interest (i.e., MSI, PEI, and SER), which will be referred to as the ADC + MSI, ADC + PEI, and ADC + SER habitats. As an example, we provide a detailed description of how we generated the habitats using MSI as our DCE quantity of interest and visualize the process in Fig. 1C. For each location that is within the tumor ROI, we constructed a vector consisting of the ADC and MSI values at V1 and V2, $\:\left\{ADC\left(\varvec{x},t=V1\right),\:ADC\left(\varvec{x},t=V2\right),\:MSI\left(\varvec{x},\:t=V1\right),\:MSI\left(\varvec{x},t=V2\right)\right\}$. This differs from previous studies in which visits are separated when forming habitats, yielding a separate habitat map for each visit^22,33^. As we will use the habitats to calibrate a biology-based model describing change in the tumor through time, we formed the habitats using the longitudinal MRI data so that the habitats capture heterogeneity in how the tumor voxels change through space and time. (This type of “longitudinal habitat analysis” is achievable due to the inter-visit registration). After building these vectors for all voxels in the tumor ROIs for all patients, we concatenated them to form a matrix. Each column of this matrix (i.e., value across all voxels) is standardized to have a mean of zero and a standard deviation of one, by subtracting the column mean and dividing by the column standard deviation.

The *n* × 4 matrix was separated into *H* habitats using the *k*-means clustering algorithm. Biological meaning is then assigned to each of the *H* habitats. To do so, we calculated the mean ADC and MSI values across all tumor bearing voxels in all patients. We then compared the mean of the V1 and V2 ADC or MSI for each habitat to the overall mean ADC or MSI. If a habitat mean is higher than the overall mean, we assign that parameter for that habitat to a label of “high”. If the habitat mean is lower than the overall mean, we assign a label of “low”. Thus, an example habitat label is “low V1 ADC, high V2 ADC, high V1 MSI, high V2 MSI”. This label indicates the example habitat was well-perfused across both visits, meaning drug could be delivered to this region effectively leading to the reduction in tumor cell number as manifested by the increase in the ADC.

We explored using anywhere from 2 to 16 habitats. We chose a maximum number of 16 habitats as there are 16 possible habitat label combinations using the assignment scheme presented in the previous paragraph. As we did not include any spatial information when forming the habitats, we assessed the spatial connectivity (i.e., how spatially contiguous each habitat is) of the habitats using two methods that both consider the 26 nearest neighbors of a central voxel of interest in a 3 × 3 × 3 cube. For the first method, we calculated the percentage of central voxels that have no neighboring voxels in the same habitat for each habitat number across the patient cohort. We refer to this metric as the “percentage of unneighbored voxels.” For the second method, we formed a spatial interaction matrix based on Wu et al.^27^ (details are provided in Supplemental S1.2 and Figure S1). Importantly, the spatial interaction matrices are formed on a patient-specific basis and larger values on the diagonal of the matrix indicate a higher degree of spatial connectivity within a habitat. Thus, we compare the diagonal values of the spatial interaction matrices across the cohort formed using our habitats to the diagonal values of spatial interaction matrices formed using randomly shuffled versions of our habitats. Specifically, we determine if the median diagonal spatial interaction index across the cohort is higher for the ADC + MSI habitats than the randomly shuffled habitats with the Wilcoxon rank sum test at a 5% significance level.

The above methods defined for the ADC + MSI data were repeated using the ADC + PEI and ADC + SER data, yielding three options for forming the 2–16 habitats.

### Biology-based model

The change of tumor cell number in space $\:\varvec{x}$ and time $\:t$, $\:{N}_{TC}(\varvec{x},t)$, was described by the reaction-diffusion-based model that was previously developed for a subset of ARTEMIS patients^8^, as presented in Eq. (7):

7 $$\:\frac{\partial\:{N}_{TC}\left(\varvec{x},t\right)}{\partial\:t}=\nabla\:\cdot\:\left(D\left(\varvec{x},t\right)\nabla\:{N}_{TC}\left(\varvec{x},t\right)\right)+\:k\left(\varvec{x}\right){N}_{TC}\left(\varvec{x},t\right)\left(1-\frac{{N}_{TC}\left(\varvec{x},t\right)}{\theta\:}\right)-\alpha\:{N}_{TC}\left(\varvec{x},t\right)C\left(x\right){\sum\:}_{i,j}{e}^{-{\beta\:}_{j}\left(t-{\tau\:}_{i,j}\right)},$$

where $\:D(\varvec{x},t)$ is the mechanically coupled cell diffusivity, $\:k\left(\varvec{x}\right)$ is the tumor cell proliferation rate at position $\:\varvec{x}$, $\:\theta\:$ is the carrying capacity, $\:\alpha\:$ is the drug efficacy, $\:C\left(\varvec{x}\right)$ is the drug concentration, $\:{\beta\:}_{j}$ is the decay rate of the $\:j$th drug, and $\:{\tau\:}_{i,j}$ is the time of the $\:i$th administration of the $\:j$th drug.

The first term on the right-hand side of Eq. (7) describes tumor cell diffusion, or movement. If $\:D(\varvec{x},t)$ is set to a constant value, we assume that diffusion is free, or not subject to mechanical forces from the surrounding tissue. Alternatively, we can set a diffusion coefficient in the absence of external force, $\:{D}_{0}$, and couple it to the surrounding tissue mechanics *via* Eq. (8):

8 $$\:D\left(\varvec{x},t\right)={D}_{0}{e}^{-\gamma\:{\sigma\:}_{vm}(\varvec{x},t)},$$

where $\:{\sigma\:}_{vm}(\varvec{x},t)$ is the von Mises stress and $\:\gamma\:$ is an empirical coupling constant. Through exponentially damping $\:{D}_{0}$ through the von Mises stress, we reduce cell mobility based on the stiffness of the surrounding tissue. To obtain the von Mises stress, we first define mechanical equilibrium as Eq. (9):

9 $$\:\nabla\:\cdot\:\varvec{\sigma\:}\left(\varvec{x},t\right)=0,$$

where $\:\varvec{\sigma\:}(\varvec{x},t)$ is the Cauchy stress tensor, which is defined in Eq. (10) under the assumption that breast tissue is linear elastic and isotropic:

10 $$\:\varvec{\sigma\:}={\uplambda\:}\left(\varvec{x}\right)\left(\nabla\:\cdot\:\varvec{u}\left(\varvec{x},t\right)\right)\mathbf{I}+\mu\:\left(\varvec{x}\right)\left(\nabla\:\varvec{u}\left(\varvec{x},t\right)+\nabla\:\varvec{u}{\left(\varvec{x},t\right)}^{\text{T}}\right)-\frac{\kappa\:{N}_{TC}(\varvec{x},t)}{\theta\:}\mathbf{I},$$

where $\:\lambda\:\left(\varvec{x}\right)=E\left(\varvec{x}\right)\nu\:/\left(\left(1+\nu\:\right)\left(1-2\nu\:\right)\right)$ and $\:\mu\:\left(\varvec{x}\right)=E\left(\varvec{x}\right)/\left(2\left(1+\nu\:\right)\right)$ are the Lamé coefficients with Young’s modulus $\:E\left(\varvec{x}\right)$ and Poisson’s ratio $\:\nu\:$, $\:\varvec{u}(\varvec{x},t)$ is the displacement, $\:\mathbf{I}$ is the second-order identity tensor, and $\:\kappa\:$ is the tumor cell-force coupling constant. With Eqs. (9) and (10), we solve for the Cauchy stress tensor $\:\varvec{\sigma\:}\left(\varvec{x},t\right)$ and subsequently calculate the von Mises stress with Eq. (11):

11 $$\begin{aligned} \sigma _{v} m(x,t) & = (1/2((\sigma _{x} x(x,t) - \sigma _{y} y(x,t))^{2} + (\sigma _{x} x(x,t) - \sigma _{z} z(x,t))^{2} + (\sigma _{z} z(x,t) - \sigma _{y} y(x,t))^{2} \\ & + 6(\sigma _{x} y(x,t)^{2} + \sigma _{x} z(x,t)^{2} + \sigma _{y} z(x,t)^{2} )))^{{(1/2)}} \\ \end{aligned}$$

We implemented the mechanical coupling as described by Hormuth et al.^42^

The second term on the right-hand side of Eq. (7) describes tumor cell proliferation through logistic growth. The proliferation rate $\:k\left(\varvec{x}\right)$ allows the tumor cells at each position $\:\varvec{x}$ to proliferate until reaching the carrying capacity, $\:\theta\:$. We calibrated the proliferation rate $\:k\left(\varvec{x}\right)$ on a patient-specific basis and calculate a patient-specific carrying capacity based on the number of spherical tumor cells with radius 10 μm that can fit within one of the patient’s imaging voxels assuming a packing density of 0.7405^39^.

The third and final term on the right-hand side of Eq. (7) describes tumor cell death due to NAT. We calibrate drug efficacy, $\:\alpha\:$, on a patient-specific basis. Drug concentration, $\:C\left(\varvec{x}\right)$ is determined using the normalized area under the DCE-MRI enhancement curve. Drug decay rates for Adriamycin and Cytoxan, $\:{\beta\:}_{1}$ and $\:{\beta\:}_{2}$, respectively, were taken from literature^43–45^. The times of the th administration of the *j*th drug, $\:{\tau\:}_{i,j}$ were provided by each patient’s treatment schedule. All fixed values and calibration ranges for this modeling framework are found in Supplemental Table S2. Through this mathematical representation of specific biological phenomena our method is immediately interpretable, as each model parameter has a known biological meaning.

### Model calibration and integration with habitat analysis

As noted above, we calibrated two parameters in Eq. (7): the proliferation rate, $\:k\left(\varvec{x}\right)$, and the drug efficacy, $\:\alpha\:$. As done with a previous model^8^, we calibrated the drug efficacy, $\:\alpha\:$, globally, meaning we obtain one value for each patient’s tumor. We explored three possible scenarios for calibrating proliferation rate $\:k\left(\varvec{x}\right)$, as seen in Fig. 1D: (1) global calibration, meaning $\:k\left(\varvec{x}\right)$ is constant for all positions $\:\varvec{x}$ in a patient’s tumor, (2) habitat-informed calibration, meaning $\:k\left(\varvec{x}\right)$ is constant for all positions $\:\varvec{x}$ within each habitat in a patient’s tumor, or (3) local calibration, meaning $\:k\left(\varvec{x}\right)$ is calibrated at each position $\:\varvec{x}$ in a patient’s tumor. Note that the third approach is what we employed in a previous study on the ARTEMIS data^8^. For all three models, we calibrated on a patient-specific basis to the V1 and V2 MRI data using the Levenberg-Marquardt algorithm^46,47^. The calibrated parameters were then used to run the model forward in time to predict tumor size, shape, and cellularity at V3. As noted in the previous section, our mathematical model is inherently interpretable, we emphasize that applying it to habitats retains the interpretability as the habitats themselves have assigned biological meaning. Furthermore, calibrating on the habitats has the added benefit of reducing the number of model parameters when compared to a local calibration.

For clarity, we emphasize that we have three habitat-informed calibrations depending on which DCE-MRE data was used to form the habitats: MSI, PEI, or SER. These calibrations will be referred to as the ADC + MSI habitat-informed calibration, ADC + PEI habitat-informed calibration, and ADC + SER habitat-informed calibration throughout the results.

### Calibration efficiency

We compared the global, habitat-informed, and local methods for calibrating $\:k\left(\varvec{x}\right)$ in terms of their calibration accuracy, prediction accuracy, and the number of parameters in each model. Models with fewer parameters generally require fewer forward runs to form the Jacobian at each iteration of the Levenberg-Marquardt algorithm and can therefore be calibrated more efficiently without increasing the efficiency of the forward run itself. To quantify this advantage, we report the number of calibrated parameters and the clock time for calibration across the patient cohort for each of the three calibration scenarios.

### Statistical methods

We defined two global metrics to evaluate calibration and prediction accuracy (Fig. 1E-F). First, we calculated the percent change in total tumor cellularity (%ΔTTC) from V1 to V2 or from V1 to V3. Total tumor cellularity at timepoint $\:t$ was calculated as the sum of voxel-wise cellularity, $\:{N}_{TC}(\varvec{x},t)$, across the tumor. Second, we calculated the percent change in total tumor volume (%ΔTTV) from V1 to V2 or from V1 to V3. The total tumor volume at timepoint $\:t$ is calculated as the number of tumor bearing voxels times the voxel volume. We calculated these metrics for every patient for each of our calibration scenarios (i.e., global, habitat-informed, or local calibration). We also calculate these metrics for the measured data at V2 and V3. We then consider the absolute difference between the calibration or prediction and data %ΔTTC or %ΔTTV for each patient.

On a voxel-by-voxel basis, we calculate the percent change in tumor cell number at each position ***x*** in the tumor ROI, %ΔTC(***x***), from V1 to V2 or from V1 to V3. We calculate %ΔTC(***x***) for the data and each of the calibration scenarios. To evaluate the voxel-by-voxel error for each calibration scenario, we calculate the mean square error between %ΔTC(***x***) on the measured data and %ΔTC(***x***) for each calibration and prediction. Thus, the final error metric, MSE(%ΔTC(***x***)), is the mean squared error between the calibrated or predicted and the measured %ΔTC(***x***).

For each calibration scenario, we consider the above three metrics (i.e., %ΔTTC, %ΔTTV, and MSE(%ΔTC(***x***))) across the patient cohort. For the three DCE-MRI quantities of interest used to form the habitats (i.e., MSI, PEI, and SER), we performed calibrations for 2–16 habitats, meaning we have 15 calibrations for per DCE-MRI quantity of interest. To determine the optimal number of habitats to use for the calibration, we take the average of each of the three error metrics at V2 and V3 for each patient, thereby yielding a distribution for each of the three error metric averages. This is done for each of the habitat numbers. We then compare the distributions at each habitat number using the two-sample Kolmogorov-Smirnov test at a 5% significance level (*kstest2*, MATLAB, R2023b; The Mathworks, Natick, MA, USA); i.e., *p* < 0.05. We define the optimal habitat number as the smallest number of habitats for which we do not find any significant difference in any of the three error distributions when comparing to all calibrations that used a larger number of habitats. As increasing the habitat number means we calibrate an additional proliferation rate, we do not view the additional proliferation rate as informative in our model if it does not offer a significant improvement in any of our error metrics.

Once the optimal habitat number is selected, we compare the global and local calibrations to the optimal habitat-informed calibrations obtained when using MSI, PEI, or SER (Fig. 1F). For these comparisons, we consider the calibration error through the distributions of the three error metrics across the cohort at V2 and the prediction error through the distributions of the three error metrics across the cohort at V3. We again use the Kolmogorov-Smirnov test at 5% significance level to evaluate for statistically significant differences between the error metrics in each comparison.

We also compared the habitat-informed, global, and local calibrations through their ability to predict pCR. To do so, the ability of the predicted and measured total tumor cellularity and total tumor volume at V3 to forecast pCR status is assessed *via* a receiver operating characteristic (ROC) curve analysis (*perfcurve*, MATLAB). The area under the ROC curve, sensitivity, and specificity were all calculated using *perfcurve* with the default settings, giving equal weight to false negative and positive misclassifications. We note that all patients received the same treatment up to V3, but that pCR status is not determined until after the second course of therapy (see Fig. 1E). Thus, to ensure consistency in patient treatments, we only considered patients who received Taxol as their second course of therapy in this part of the analysis. The AUCs for the different calibration options were compared using DeLong’s Test with *p* < 0.05 taken as the threshold for significance^48^.

## Results

### Habitat formation

**Fig. 2:**
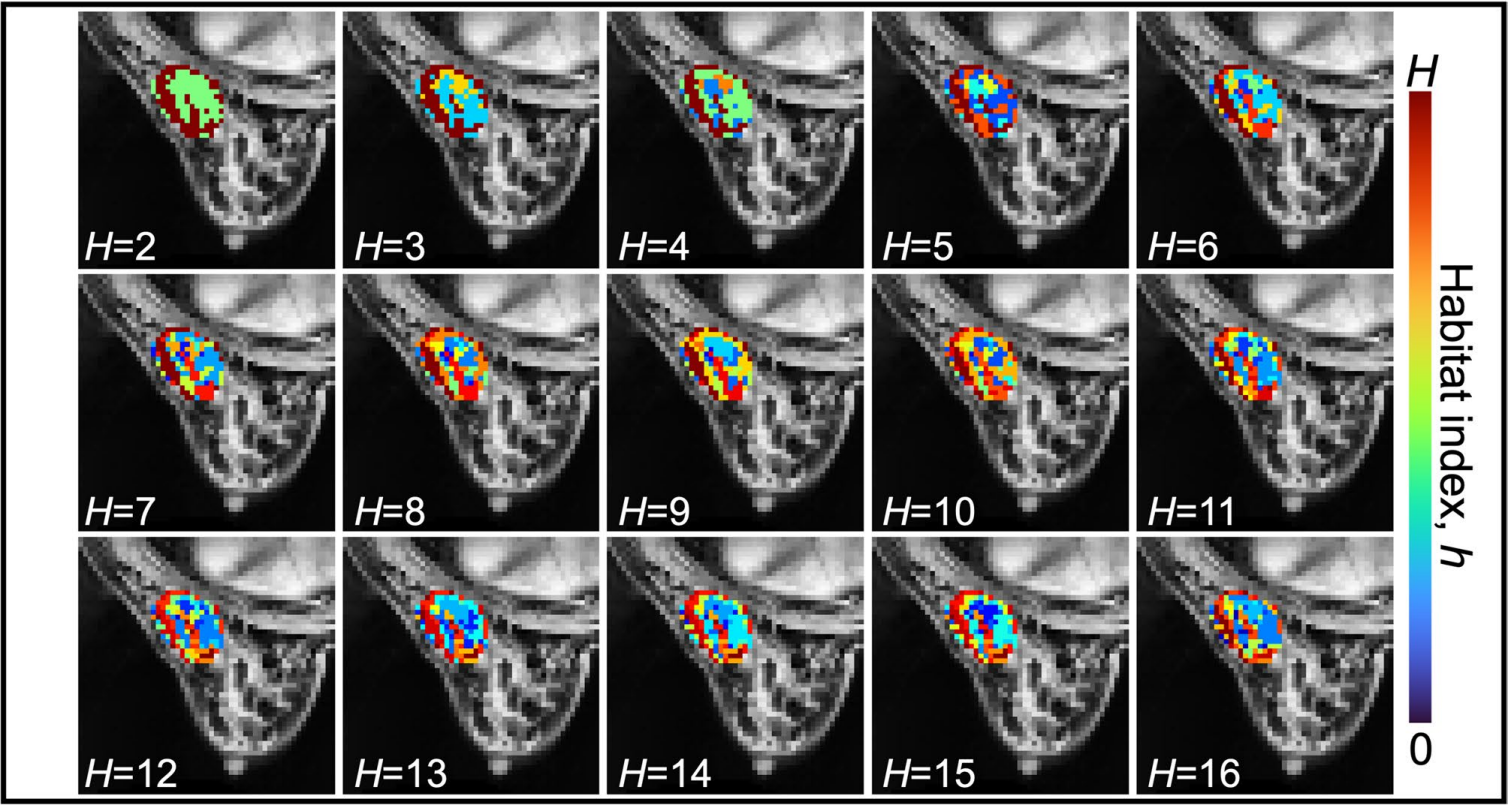
The habitats (from *H* = 2 to *H* = 16) formed over the patient cohort using the apparent diffusion coefficient (ADC) and maximum slope of increase (MSI) at V1 and V2 are visualized on the center axial tumor slice of an example patient. As the indices assigned by *k*-means clustering are random, we order the indices in terms of increasing mean V1 ADC to more easily visualize changes in the maps as we increase *H*. While the habitats are not formed using any spatial information, note that they do seem to be spatially connected. As we increase *h*, the resulting habitats capture increasing intra-tumoral heterogeneity. For example, when *H* = 2, 3, or 4 we identify a habitat covering much of the edge of the tumor on the left side of this slice. Investigating the same area with a larger *H*, such as 16, the habitat covering the left edge of the tumor is divided into additional habitats. As the habitats are used to inform regions in which a tumor cell proliferation rate is locally calibrated, the additional heterogeneity captured with increasing *H* can also result in more heterogeneity in the calibrated proliferation rates.

Figure 2 shows center slice maps for *H* = 2–16 habitats generated using the ADC + MSI data for an example patient. (Supplemental Figures S2 and S3 shows the same habitats generated when using the ADC + PEI data and the ADC + SER data, respectively). Visually, the habitats appear to have a degree of spatial connectivity which we quantified by finding the percentages of lonely voxels within each habitat. For example, when the ADC + PEI data were used to generate two habitats, those percentages were only 0.041% and 0.31% for habitats 1 and 2, respectively, with similar values of 0.072% and 0.24% for habitats 1 and 2, respectively, generated from the ADC + MSI data and 0.12% and 0.26% for habitats 1 and 2, respectively, generated from the ADC + SER data. At 16 habitats, we found median (range) of percent lonely voxels across the 16 habitats of 3.6 (0.38–6.8) % for ADC + PEI data, 3.2 (0.23–5.3) % for ADC + MSI data, and 3.9 (1.7–5.9) % for ADC + SER data. The maximum percent of lonely voxels for any habitat across all data types was 6.8%, which was found for one of 16 habitats generated by the ADC + PEI data. We further examined spatial connectivity in our habitats using the diagonals of the spatial interaction matrices, where larger values indicate greater spatial connectivity within each habitat^27^. For all 2–16 habitats constructed using each of the three data options, we found that the median diagonal spatial interaction index across the cohort of the ADC + MSI habitat maps is significantly higher than the median diagonal spatial interaction index of randomly shuffled habitats. For example, for the *H* = 3 habitats generated with ADC + MSI data, the median (IQR) diagonal spatial interaction index across the cohort was 0.23 (0.088, 0.46), while the median (IQR) for the randomly shuffled habitats was 0.14 (0.038, 0.36). These medians are found to be significantly different with the Wilcoxon rank sum test (*p* < 0.05). Further results on spatial connectivity are in Supplemental S2.1 and Supplementary Material 4.

### Selecting the optimal habitat number

To determine the optimal number of habitats to use for the calibration, we computed the average of each of the three error metrics (i.e., %ΔTTC, %ΔTTV, and MSE(%ΔTC(***x***))) at V2 and V3 for each patient. Then we took the resulting distributions of these metrics across the patient cohort and compared them across the habitat numbers using the two-sample Kolmogorov-Smirnov test at a 5% significance level. We found that the error distributions for three habitats generated from the ADC + MSI, ADC + PEI, and ADC + SER data sets did not show any significant differences when compared to the error distributions for four or more habitats. Thus, we selected three habitats as the most parsimonious and all analyses reported below are only for this optimal habitat number. (All *p*-values for these comparisons are found in Supplemental S2.2, Supplementary Material 1.)

### Representative patients

**Fig. 3:**
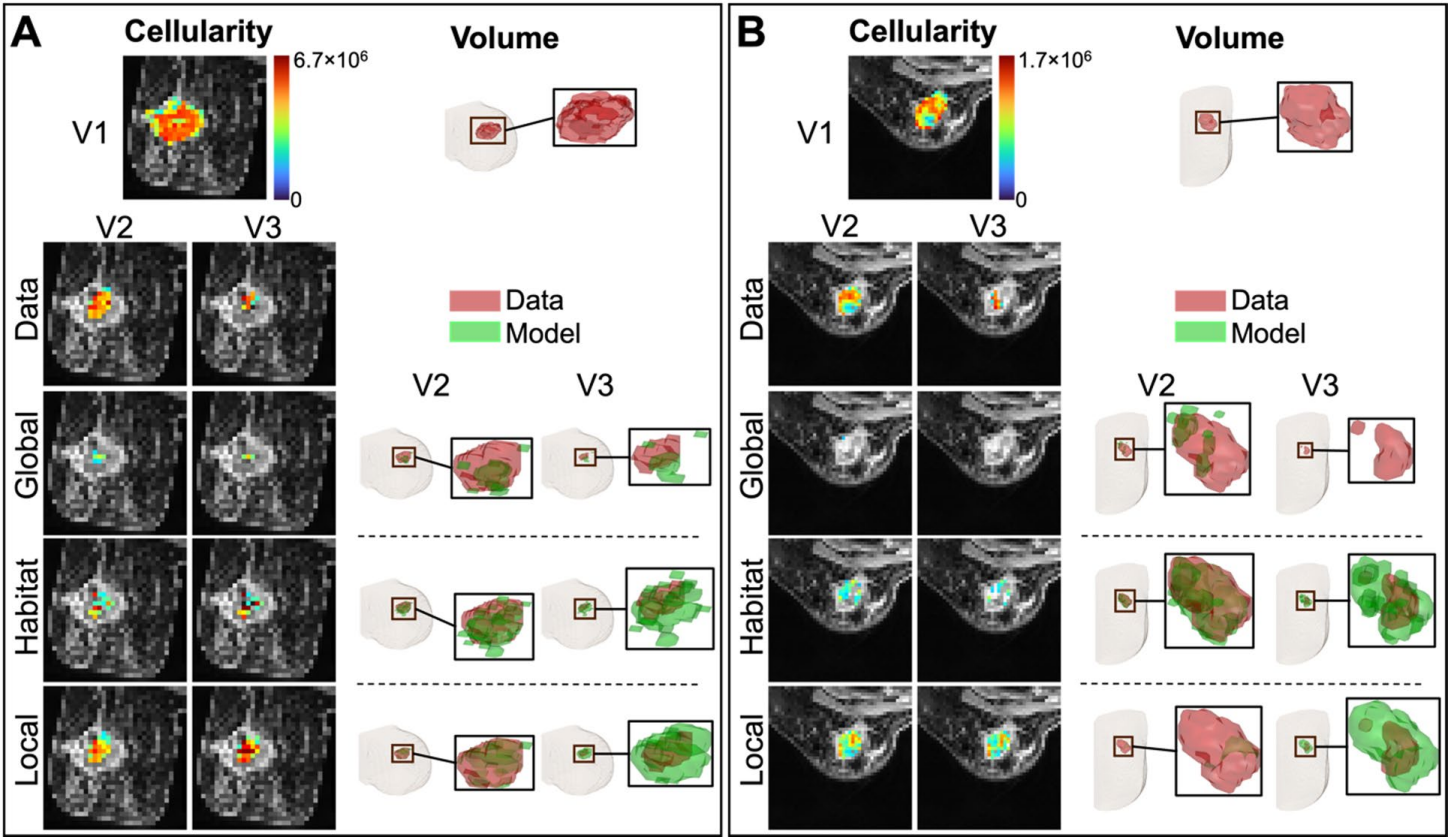
Panel (**A**) shows a representative pCR patient, selected as the patient with median average error in TTC at V2 and V3 from the ADC + MSI habitat-informed calibration across the cohort of pCR patients. The left column of the panel presents, the center slice from visits V2 and V3 for the measured MRI data, as well as the global, ADC + MSI, and local calibrations. At V2, the timepoint used for calibration, the absolute difference between the modeled and measured cellularity, %DTTC, is 21%, 14%, and 6.7% for the global, habitat-informed, and local calibrations, respectively. At V3, the timepoint used for prediction, the %DTTC is 3.7%, 3.6%, and 13% for the global, habitat, and local predictions, respectively. Tumor volume is visualized on the right of the panel, with the absolute difference between modeled and measured, %DTTV, at V2 being 20%, 12%, and 4.6% for the global, habitat, and local calibrations, respectively. At V3, we obtained %DTTV of 2.8%, 4.7%, and 13% for the global, habitat, and local predictions, respectively. For a representative non-pCR patient, shown in Panel (**B**) the %DTTC at V2 was 29%, 15%, and 4.3% for the global, habitat, and local calibrations, respectively. At V3, %DTTC was 5.4%, 3.8%, and 18%, respectively. Looking at the error in tumor volume, we obtained %DTTV of 31%, 10%, and 1.1% at V2 and 5.9%, 8.7%, and 24% at V3 for the global, habitat, and local calibrations, respectively.

Panels A and B of Fig. 3 present the error in tumor cellularity and volume for the global, ADC + MSI habitat-informed, and local calibrations for a representative pCR patient and a representative non-pCR patient, respectively. (To select a representative patient, we averaged the ADC + MSI habitat-informed calibration (V2) and prediction (V3) error in %ΔTTC for each patient and selected the patient with median error in each of the pCR and non-pCR patient cohorts.) In both panel A and B, the first row shows the V1 initial condition used for all calibrations, while the second row shows the V2 and V3 cellularity as measured from the DW-MRI data. The third through fifth rows show the modeled cellularity and volume for a globally, habitat, and locally calibrated proliferation rate and a globally calibrated drug efficacy.

For the representative pCR patient in Fig. 3A, the absolute difference between the measured and V2 calibrated cellularity (i.e., %ΔTTC) is 21%, 14%, and 6.7% for the global, habitat-informed, and local calibrations, respectively. When analyzing the %ΔTTC predicted for V3, we obtain 3.7%, 3.6%, and 13% for the global, habitat-informed, and local predictions, respectively. Then, considering the local cellularity, the mean square error between the measured and V2 calibrated %ΔTC(***x***) is 0.14, 0.11, and 0.013 for the global, habitat-informed, and local calibration, respectively. Repeating this for the V3 predictions gives 0.032, 0.078, and 0.15, respectively. The absolute difference between the measured and V2 calibrated tumor volume (i.e., %ΔTTV) is 20%, 12%, and 4.6% for the global, habitat-informed, and local calibrations, respectively. For the %ΔTTV predicted at V3, we obtain 2.8%, 4.7%, and 13%, respectively.

For the representative non-pCR patient in Fig. 3B, the absolute difference between the measured and V2 calibrated cellularity (i.e., %ΔTTC) is 29%, 15%, and 4.3% for the global, habitat-informed, and local calibrations, respectively. When analyzing the %ΔTTC predicted for V3, we obtain 5.4%, 3.8%, and 18%, for the global, habitat-informed, and local predictions, respectively. Then, considering local cellularity, the mean square error between the measured and V2 calibrated %ΔTC(***x***) is 0.29, 0.19, and 0.0092 for the global, habitat-informed, and local calibration, respectively. Repeating this for the V3 predictions gives 0.078, 0.12, and 0.22, respectively. The absolute difference between the measured and V2 calibrated tumor volume (i.e., %ΔTTV) is 31%, 10%, and 1.1% for the global, habitat-informed, and local calibrations, respectively. For the, %ΔTTV predicted at V3, we obtain 5.9%, 8.7%, and 24%, respectively.

### Comparison of global, habitat, and local calibration/prediction accuracy

**Fig. 4:**
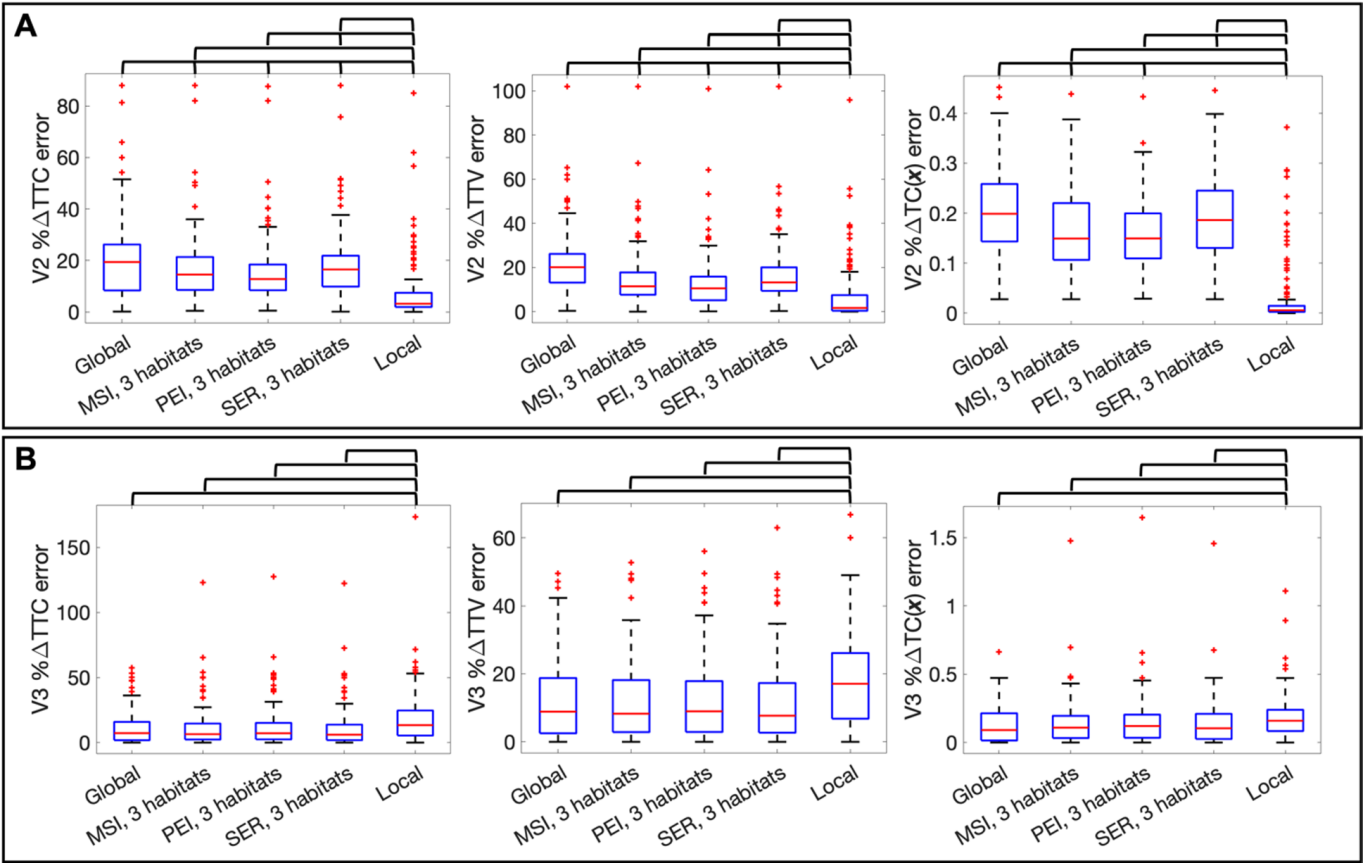
Panel (**A**) shows the %DTTC, %DTTV and %DTC(***x***) errors for the calibration from V1 to V2. The bars above each plot indicate a significant difference between each pair of error distributions by the two-sample Kolmogorov-Smirnov test at a 5% significance level. Note that the local calibration error is always significantly lower than the other calibration schemes. The global calibration error is higher than all habitat-informed calibrations apart from the %DTC(***x***) error for the ADC + SER habitat-informed calibration. There is no significant difference between the ADC + PEI and ADC + MSI habitat-informed calibration errors. The ADC + SER and ADC + MSI habitat-informed calibration errors also do not show significant differences. The ADC + SER habitat-informed calibration error is higher than the ADC + PEI habitat-informed calibration error in all cases. Panel (**B**) shows the same three errors for the model predictions to V3. Here, we do not find significant differences between any combination of predictions from the global, ADC + MSI, ADC + PEI, and ADC + SER habitat-informed calibrations for any error metric. Predictions from the global, ADC + MSI, ADC + PEI, and ADC + SER habitat-informed calibrations all have significantly lower error than the predictions from the locally calibration for all three error metrics.

We now consider calibration error defines as the error in %ΔTTC, %ΔTTV, and %ΔTC(***x***) at V2, across the patient cohort. The boxplots in Fig. 4 compare the global, ADC + MSI habitat-informed, ADC + PEI habitat-informed, ADC + SER habitat-informed, and local calibration errors in %ΔTTC, %ΔTTV, and %ΔTC(***x***). The median (interquartile range) of each of these errors for each visit is found in Table 1.

**Table 1:** Calibration and prediction errors across models.

Calibration	V2 cohort median (IQR) error in:			V3 cohort median (IQR) error in:		
	Total tumor cellularity	Total tumor volume	Local cellularity	Total tumor cellularity	Total tumor volume	Local cellularity
Global	19 (8.4–26)%	20 (13–26)%	0.20 (0.14–0.26)	7.2 (1.8–16)%	8.9 (2.5–19)%	0.091 (0.015–0.21)
ADC + MSI	15 (8.5–21)%	11 (7.6–18)%	0.15 (0.11–0.22)	6.4 (2.3–15)%	8.3 (2.9–18)%	0.11 (0.033–0.19)
ADC + PEI	13 (8.4–18)%	11 (5–16)%	0.15 (0.11–0.20)	7.2 (2.5–15)%	9.0 (2.9–18)%	0.12 (0.034–0.20.034.20)
ADC + SER	17 (9.8–22)%	13 (9.4–20)%	0.19 (0.13–0.25)	6.0 (1.8–14)%	7.7 (2.7–17)%	0.10 (0.026–0.21)
Local	3.1 (1.9–7.4)%	1.7 (0.41–7.5)%	0.0058 (0.0027–0.015)	13.4 (5.4–25)%	17 (6.8–26)%	0.16 (0.083–0.24)

V2 = visit 2, after two cycles of NAT.

V3 = visit 3, after four cycles of NAT.

ADC = apparent diffusion coefficient.

MSI = maximum slope of increase.

PEI = positive enhancement integral.

SER = signal enhancement ratio.

Figure 4A depicts the distributions of absolute difference between the calibrated and measured %ΔTTC, absolute difference between the calibrated and measured %ΔTTV, and the mean square error between the calibrated and measured %ΔTC(***x***) across the patient cohort. First, we consider the absolute difference between the calibrated and measured %ΔTTC from V1 to V2. The local calibration has a significantly lower error than all other calibrations with a median (IQR) error of 3.1 (1.9–7.4)%. The global calibration has a significantly higher error than all other calibrations, with and error of 19 (8.4–26)%. When using the ADC + MSI, ADC + PEI, and ADC + SER data sets to identify the three habitats, the calibrations have errors of 15 (8.5–21)%, 13 (8.4–18)%, and 17 (9.8–22)%, respectively. Between these calibrations, we only find significant differences between the calibrations with the ADC + PEI and ADC + SER habitat-informed calibrations. Considering the absolute difference between the calibrated and measured %ΔTTV, we again find that the local calibration has significantly lower error than the other calibrations with 1.7 (0.41–7.5)%, while the global calibration has significantly higher error of 20 (13–26)%. The ADC + MSI, ADC + PEI, and ADC + SER habitat-informed calibrations have median (IQR) values of 11 (7.6–18)%, 11 (5.2–16)%, and 13 (9.4–20)%, respectively. We again only find a significant difference between the ADC + PEI and ADC + SER calibrations. For the mean square error between the calibrated and measured %ΔTC(***x***), we find that the local calibration has a significantly lower error than the other calibrations with a median (IQR) of 0.0058 (0.0027–0.015). The global calibration has a significantly higher error than the ADC + MSI and ADC + PEI habitat-informed calibrations at 0.20 (0.14–0.26), 0.15 (0.11–0.22), and 0.15 (0.11–0.20), respectively. We found no significant difference between the global calibration and the ADC + SER habitat-informed calibrations, which has an error of 0.19 (0.13–0.25). Between the habitat-informed calibrations, we again only find a significant difference between the ADC + PEI and ADC + SER habitat-informed calibrations. All *p*-values for these comparisons are found in Supplemental S2.3.

In Fig. 4B, we repeat the same error plots as Fig. 4A, but for the predictions to V3. First, we consider the absolute difference between the predicted and measured %ΔTTC from V1 to V3. The prediction from the local calibration has significantly higher error than all other predictions with a median (IQR) error of 13 (5.4–25)%. When using a global proliferation rate or a habitat-informed proliferation rate from the ADC + MSI, ADC + PEI, and ADC + SER data, the predictions have errors of 7.2 (1.8–16)%, 6.4 (2.3–15)%, 7.2 (2.5–15)%, and 6.0 (1.8–14)%, respectively. Amongst these four predictions, we do not find any significant difference in the errors. Considering the absolute difference between the predicted and measured %ΔTTV, we again find that the prediction from the local calibration has significantly higher error than the other calibration scenarios with 17 (6.8–26)%. When using a global proliferation rate or habitat-informed proliferation rate from the ADC + MSI, ADC + PEI, and ADC + SER data, the predictions have errors of 8.9 (2.5–19)%, 8.3 (2.9–18)%, 9.0 (2.9–18)%, 7.7 (2.7–17)%, respectively. We again do not find any significant difference in the errors. For the mean square error between the predicted and measured %ΔTC(***x***), we find that the prediction from the local calibration has a significantly higher error than the predictions from the global or habitat-informed calibrations with a median (IQR) of 0.16 (0.083–0.24). When using a global proliferation rate or a proliferation rate informed by the three habitats formed using ADC + MSI, ADC + PEI, and ADC + SER data, the predictions have errors of 0.091 (0.015–0.21), 0.11 (0.033–0.19), 0.12 (0.034–0.20.034.20), and 0.10 (0.026–0.21), respectively. All *p*-values for these comparisons are found in Supplemental S2.3, Supplementary Material 3.

### Comparison of global, habitat, and local calibration efficiency

In addition to calibration accuracy, we considered calibration efficiency through the number of parameters in each of the global, habitat-informed, and local calibrations. For the global calibration, we had single proliferation rate and drug efficacy value for each tumor, so we calibrated only two parameters for each patient. This results in a median (range) wall clock time to calibrate of 9.8 (1.0–56) minutes across the patient cohort. For the habitat-informed calibration with our optimal habitat number of three, we had three proliferation rates (i.e., one for each habitat) and a single drug efficacy value, so we calibrated four parameters for each patient. This results in a median (range) time to calibrate of 9.1 (0.56–40) minutes across the patient cohort when using the ADC + MSI habitats. For the local calibration, we had a single drug efficacy value and a proliferation rate for each voxel in their tumor which resulted in a median (range) of 648 (52 − 3,809) parameters to be calibrated. This results in a median (range) time to calibrate of 153 (5.9–2,100) minutes across the patient cohort. All calibrations were performed on an AMD EPYC 7402P (2.8 GHz) CPU with 24 cores, 48 threads, and 128 GB of memory.

### Predicting pCR

**Fig. 5:**
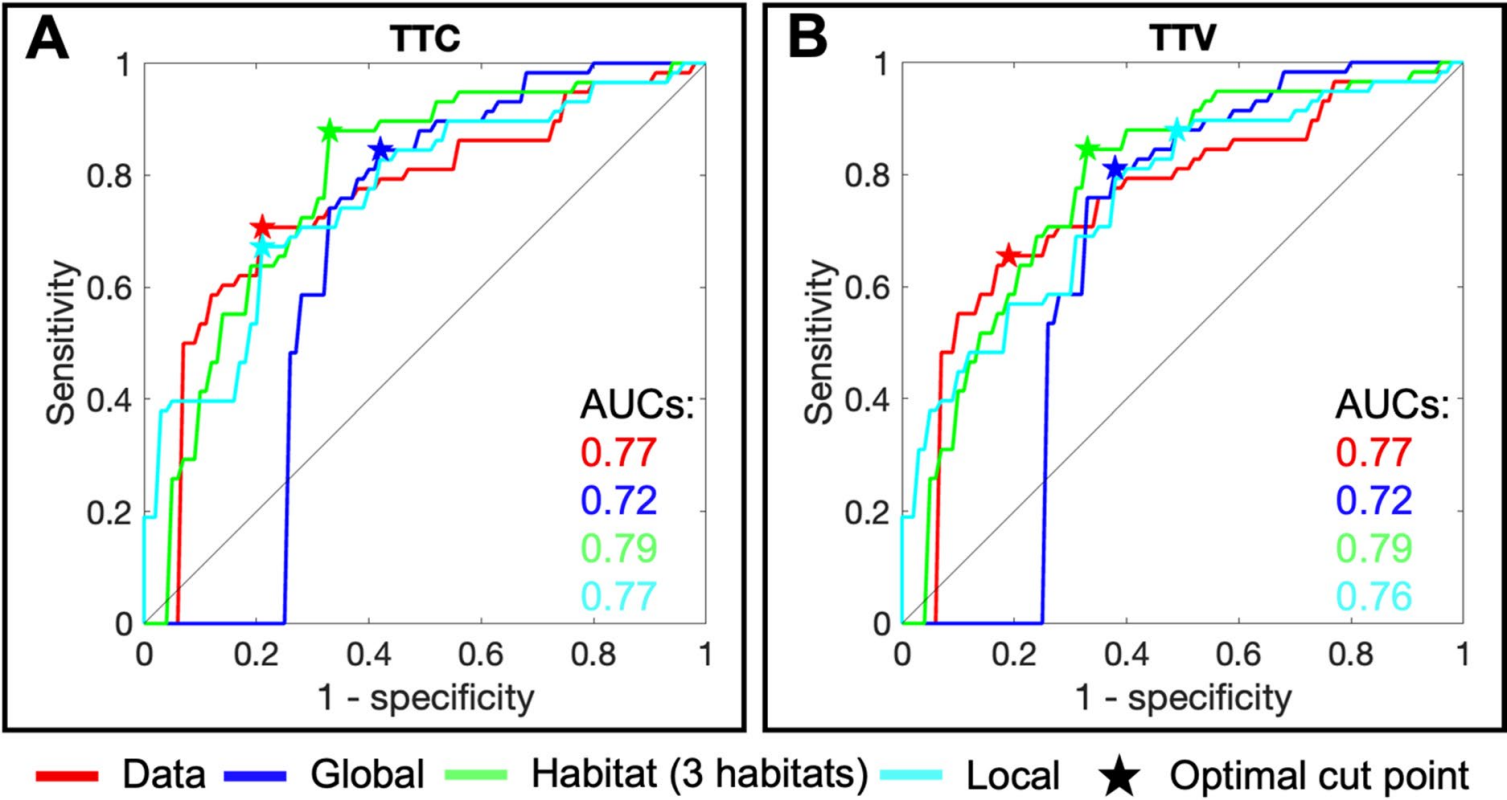
Panel (**A**) shows the ROC curves for predicting pCR status using the V3 total tumor cellularity (TTC). The red curve is from the TTC measured from the V3 data. The blue, green, and teal curves are from the V3 TTC predicted from calibrations using V1 and V2 data with a global, ADC + MSI habitat-informed, or local proliferation rate, respectively. We obtained AUC (95% CI) values of of 0.77 (0.67–0.86), 0.72 (0.61–0.82), 0.79 (0.68–0.87), and 0.77 (0.65–0.85), respectively. Using DeLong’s test, we do not find any significant difference between these AUC values. Panel (**B**) shows the ROC curves for predicting pCR status using the V3 total tumor volume (TTV). The red curve is from the TTV measured from the V3 data. The blue, green, and teal curves are from the V3 TTV predicted from calibrations using V1 and V2 data with a global, ADC + MSI habitat-informed, or local proliferation rate, respectively. We obtained AUC values of 0.77 (0.67–0.85), 0.72 (0.62–0.82), 0.79 (0.67–0.87), and 0.76 (0.66–0.84), respectively. Using DeLong’s test, we do not find any significant difference between these AUC values. Overall, we do not find that the AUC values from the predictions using any of the model calibration options (i.e., global, habitat-informed, or local proliferation rates) are significantly different, and we do not find that these AUC values are significantly lower than the AUC values from the measured data. Thus, we can predict pCR from V1-V2 calibrations without significantly reducing AUC from pCR predictions using measured V3 data. In particular, this means calibrating a biology-based model to a limited number of habitats allows for accurate and interpretable predictions of patient response to NAT.

Figure 5 presents the ROC analysis in which we use the measured and predicted (from the global, ADC + MSI habitat-informed, and local calibrations) total tumor cellularity and volume at V3 as predictors of pCR status. Figure 5A shows the ROC curve analysis for total tumor cellularity. We obtain AUC (95% CI) values of 0.77 (0.67–0.86), 0.72 (0.61–0.82), 0.79 (0.68–0.87), and 0.77 (0.65–0.85) for the measured data and global, ADC + MSI habitat-informed, and local calibrations, respectively. We do not find a significant difference between these AUC values. Figure 5B shows the ROC curve analysis for total tumor cellularity. We obtain AUC (95% CI) values of 0.77 (0.67–0.85), 0.72 (0.62–0.82), 0.79 (0.67–0.87), and 0.76 (0.66–0.84) for the measured data and global, ADC + MSI habitat-informed, and local calibrations, respectively. While the habitat-informed predictions do have the highest AUC values, we note that we do not find a significant difference between these AUC values. Table 2 presents all AUC, sensitivity, and specificity values from this analysis and the analyses for the ADC + PEI and ADC + SER calibrations. All *p*-values for AUC comparisons and ROC curve plots for the pCR predictions from the ADC + PEI and ADC + SER habitat-informed calibrations are found in Supplemental S2.4 (Supplementary Material 2) and Figures S5 and S6. We also explore the direct prediction of pCR status from the percent of tumor volume in each habitat in Supplemental S2.2 and Figure S4.

**Table 2:** Results of ROC curve analyses for predicting pCR status.

Predictor	Calibration to obtain predictor	AUC (95% CI)	Specificity	Sensitivity
**V3 total tumor cellularity**	Data	0.77 (0.67–0.86)	0.79	0.71
	Global	0.72 (0.61–0.82)	0.58	0.84
	ADC + MSI habitat	0.79 (0.68–0.87)	0.67	0.88
	ADC + PEI habitat	0.78 (0.66–0.86)	0.69	0.84
	ADC + SER habitat	0.77 (0.67–0.85)	0.58	0.88
	Local	0.77 (0.65–0.85)	0.79	0.67
**V3 total tumor volume**	Data	0.77 (0.67–0.85)	0.81	0.66
	Global	0.72 (0.62–0.82)	0.62	0.81
	ADC + MSI habitat	0.79 (0.67–0.87)	0.67	0.84
	ADC + PEI habitat	0.78 (0.67–0.87)	0.72	0.81
	ADC + SER habitat	0.78 (0.66–0.86)	0.55	0.93
	Local	0.76 (0.66–0.84)	0.51	0.88

AUC = area under receiver operating characteristic curve.

V3 = visit 3, after four cycles of NAT.

ADC = apparent diffusion coefficient.

MSI = maximum slope of increase.

PEI = positive enhancement integral.

SER = signal enhancement ratio.

### Assigning meaning to habitats

**Fig. 6:**
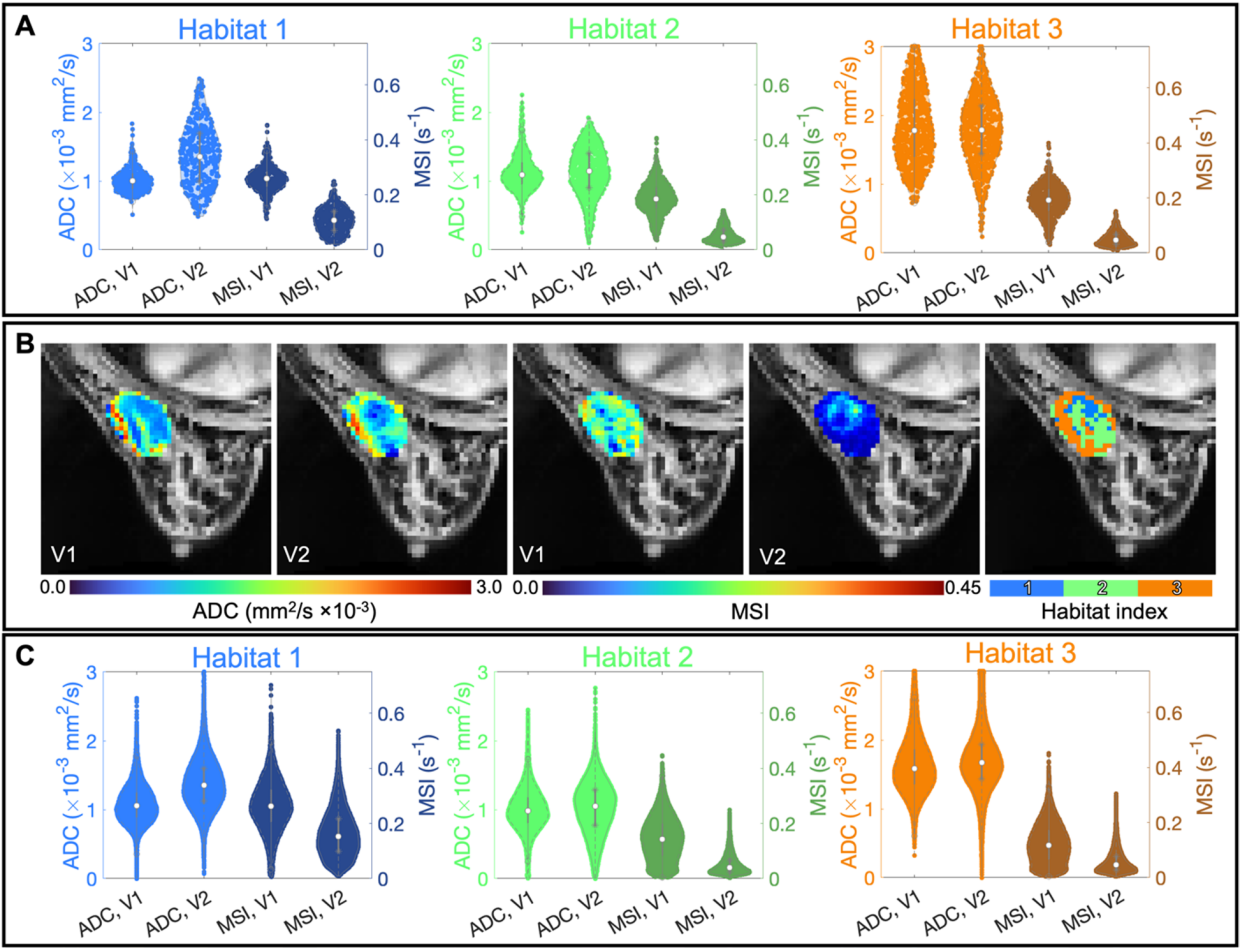
Panel (**A**) shows distributions of ADC and MSI at V1 and V2 for each of the three habitats for an example patient. Panel (**B**) shows ADC, MSI, and habitat for the center slice of the same patient’s tumor. Panel **C** has the same plots as Panel A, but for all tumor bearing voxels across all patients. We use the mean values of each parameter distribution in each habitat to assign habitat meanings. The overall mean ADC and MSI values are 1.3 × 10^− 3^ mm^2^/s and 0.12 s^− 1^, respectively. For habitat 1, the mean ADC value at V1 is lower than the overall mean, and the mean ADC value at V2 is higher than the overall mean. Thus, this habitat has high cellularity at V1 and low cellularity at V2. Using the same logic for the habitat 1 MSI, we assign the label of high V1 and V2 MSI. Taken in concert, habitat 1 is interpreted as a region that is well-perfused and capable of delivering treatment to the tumor effectively (as manifested by the high MSI value at both V1 and V2) which can then effectively kill tumor cells (as manifested by the decrease in cellularity from V1 to V2). Habitat 2 has low V1 and V2 ADC, corresponding to high V1 and V2 cellularity. When combined with the high V1 and low V2 MSI, we interpret habitat 2 as experiencing a reduction in perfusion from V1 to V2, meaning it is increasingly difficult to deliver treatment to this region. As a result, fewer tumor cells are killed, and the cellularity remains high at V2. Habitat 3 has high V1 and V2 ADC, meaning cellularity is consistently low in this habitat. With a low MSI across visits, this habitat is interpreted as poorly perfused, meaning both drug and nutrients may not be effectively delivered to this habitat. Thus, the cellularity remains consistently low, as the tumor is not able to grow or die due to lack of nutrient and drug delivery, respectively.

Figure 6 presents the distributions of each of the quantities of interest used to form the ADC + MSI habitats. These distributions allowed us to assign biological meaning to each of the habitats. Panels A and B illustrate the ADC and MSI as distributions and center slice maps for an example patient. In Fig. 6A, violin plots show the distributions of ADC at V1 and V2 and MSI at V1 and V2 for each of the three habitats for an example patient. For this patient, habitat 1 has mean (standard deviation) ADC values at V1 and V2 of 1.0 (0.17)×10^− 3^ and 1.4 (0.44)×10^− 3^ mm^2^/s, respectively, and mean MSI values at V1 and V2 of 0.26 (0.051) and 0.11 (0.047) s^− 1^, respectively. Habitat 2 has ADC values of at V1 and V2 of 1.1 (0.27)×10^− 3^ and 1.1 (0.36)×10^− 3^ mm^2^/s, respectively, and mean MSI values at V1 and V2 of 0.19 (0.059) and 0.054 (0.029) s^− 1^, respectively. Habitat 3 has ADC values at V1 and V2 of 1.8 (0.54)×10^− 3^ and 1.8 (0.52)×10^− 3^ mm^2^/s, respectively, and MSI values at V1 and V2 of 0.19 (0.059) and 0.051 (0.026) s^− 1^, respectively. Figure 6B shows ADC maps at V1 and V2, MSI maps at V1 and V2, and the resulting habitat map for the center slice of the example patient.

Figure 6C contains the same plots as Fig. 6A. but for all voxels across the entire patient cohort. We used these plots to assign cohort-wide biological meaning to the habitats. Across V1 and V2 over the entire cohort, the mean (standard deviation) ADC is 1.3 (0.47)×10^− 3^ mm^2^/s and the mean MSI value is 0.12 (0.095) s^− 1^. For habitat 1, the ADC values at V1 and V2 were 1.1 (0.27)×10^− 3^ and 1.4 (0.36)×10^− 3^ mm^2^/s, respectively. Compared to the overall mean ADC, this corresponds to a low V1 and high V2 ADC or, equivalently, high V1 and low V2 cellularity. The MSI values at V1 and V2 were 0.26 (0.091) and 0.16 (0.084) s^− 1^, respectively. Compared to the overall mean MSI, this corresponds to a high V1 and V2 MSI. With a high MSI at V1 and V2, we interpret habitat 1 as being a well-perfused region of the tumor. Thus, drug was delivered to this region of the tumor effectively, leading to the reduction in tumor cellularity indicated by the decrease in cellularity between V1 and V2.

For habitat 2, the mean ADC values at V1 and V2 were 0.99 (0.31)×10^− 3^ and 1.0 (0.38)×10^− 3^ mm^2^/s, respectively. Compared to the overall mean ADC, this habitat had low V1 and low V2 ADC, corresponding to high V1 and V2 cellularity. The MSI values at V1 and V2 were 0.14 (0.078) and 0.051 (0.035) s^− 1^, respectively. Compared to the overall mean MSI, this corresponds to a high V1 and low V2 MSI. With MSI decreasing from V1 to V2, we interpret habitat 2 as becoming less well-perfused in time. Thus, less drug was delivered to the tumor indicating the tumor cell number remained high from V1 to V2.

For habitat 3, the mean ADC values at V1 and V2 were 1.6 (0.40)×10^− 3^ and 1.7 (0.44)×10^− 3^ mm^2^/s, respectively. Compared to the overall mean ADC, this habitat had high V1 and high V2 ADC, corresponding to low V1 and V2 cellularity. The MSI values at V1 and V2 were 0.12 (0.071) and 0.058 (0.040) s^− 1^, respectively. Compared to the overall mean MSI, this habitat had low V1 and low V2 MSI. With low MSI values at both visits, we interpreted habitat 2 as a poorly perfused region of the tumor to which neither nutrients nor drugs could be easily delivered. Thus, tumor cells could not effectively grow (due to lack of nutrients) or die (due to lack of drugs), resulting in low cellularity at V1 and V2.

Results for the parameter values calibrated to each habitat to confirm consistency in calibrated parameters between the local and habitat calibrations are found in Supplemental S2.5 and Figure S7.

## Discussion

We have shown that by integrating biology-based modeling and habitat analysis, we can predict TNBC response to neoadjuvant therapy with a significantly higher accuracy than models calibrated at the voxel level. Importantly, by using habitats to inform the biology-based model calibration, we retain the interpretability of the biology-based model that is not possible when directly making tumor response predictions from the habitats themselves. The habitats also reduce the number of parameters required to calibrate the model as compared to the local calibration by a factor of 162 (4 versus a median (range) of 648 (52 − 3,809) parameters, respectively), meaning the time to calibrate can be reduced by a factor of 17 (taking a median (range) of 9.1 (0.56–40) minutes versus 153 (5.9–2,100) minutes, respectively). Overall, integrating habitats and biology-based modeling addresses the need to predict TNBC patient response to NAT in an accurate, interpretable, and efficient manner.

To evaluate the habitat-informed calibrations, we first investigated the calibration accuracy for each of the habitat-informed calibrations (i.e., ADC + MSI, ADC + PEI, and ADC + SER), as well as the global and local calibrations. When comparing the three habitat-informed calibrations, we found that the ADC + PEI habitat informed calibration had significantly lower error on all metrics (i.e., %ΔTTC, %ΔTTV, and MSE(%ΔTC(***x***)) than the ADC + SER habitat informed calibration. We found no other significant differences between the habitat-informed calibrations. This could result from the fact that the PEI integrates across all frames of the DCE-MRI acquisition timecourse, while the SER is calculated using only three frames, meaning the ADC + PEI habitats contain more information from the DCE-MRI timecourse. When comparing the global, habitat-informed, and local calibrations for or our global evaluation metrics (%ΔTTC and %ΔTTV), we found that the global calibration has significantly higher error and the local calibration has significantly lower error than the habitat-informed calibrations for both metrics. This is expected, as increasing the number of parameters allows for a better fit to the data. For the local cellularity metric, %ΔTC(***x***), at V2, there was not a significant difference between the calibration accuracy obtained by the global and ADC + SER habitat-informed calibrations, while the global calibration error was significantly higher than the ADC + MSI and ADC + PEI habitat-informed calibrations.

Next, we investigated the prediction accuracy at V3. Across all three error metrics, we did not find any significant differences between any combinations of the predictions obtained from the global, ADC + MSI, ADC + PEI, or ADC + SER habitat-informed calibrations. The prediction from the local calibration, however, had significantly higher error than the predictions from the global and all three habitat-informed calibrations for all three metrics, suggesting that the local calibration may have overfit the data. Thus, we conclude that the local calibration provides the least accurate predictions, and the global and habitat-informed calibrations all provide statistically equivalent prediction accuracy.

Finally, we performed the ROC curve analysis in which we use the total tumor cellularity and volume predicted at V3 to predict pCR status at the time of surgery. Using DeLong’s test, we did not find a significant difference between AUC values obtained by the predictions of any pair of calibration options. While we use V3 cellularity and volume as predictors of pCR here, we note that all patients underwent a second regimen of NAT (i.e., 12 weekly cycles of Taxol) before the tumor was resected and pCR was determined. We also computed the AUC values from the measured data at V3 and did not find a statistically significant difference between the AUCs computed from the data compared to any of our model calibration options. Thus, we conclude that none of our calibrations perform statistically worse than the data.

Overall, when considering calibration error at V2, the ADC + MSI and ADC + PEI habitat-informed calibrations have significantly lower error than the global calibration and higher error than the local calibration. When making predictions to V3, the ADC + MSI habitat and ADC + PEI habitat-informed predictions have significantly lower error than the locally calibrated predictions and no significant difference form the globally calibrated predictions. Thus, the ADC + MSI habitat and ADC + PEI habitat-informed calibrations offer the best balance between the calibration and prediction accuracy.

One important distinction between this work and previous habitat analysis studies is our use of multiple timepoints to form longitudinal habitats capturing changes in the tumor, rather than forming habitats separately at multiple timepoints for longitudinal data^22,23,29,33^, or at a single timepoint^24–26,28^. For example, Carvalho et al.^29^. observed that changes in their habitats through time could be indicative of pCR for breast cancer patients, motivating our goal to capture change within the habitats. As the habitats in our analysis were used to inform the calibration of model parameters describing dynamics of tumor response to NAT, forming habitats with longitudinal data ensured that the habitats would capture regions of the tumor that experience similar changes in time, rather than capturing regions with distinct characteristics at a particular point in time.

Wu et al.^8^. used a similar biology-based modeling framework with a locally calibrated proliferation rate on a subset of 56 patients from the ARTEMIS trial. To predict pCR status, their model is calibrated to V1, V2, and V3 data, then run forward to the timepoint of surgery to obtain total tumor cellularity and volume values that were used as predictors of pCR status, reporting AUC values of 0.89 for both^8^. In the current study, we obtained AUC values of 0.79 from the cellularity and volume predicted by the ADC + MSI model. While this is a lower AUC value, we obtained this prediction using only V1 and V2 data. Additionally, the model presented by Wu et al.. uses the locally calibrated proliferation rate presented here, with hundreds of parameters calibrated to the model for each patient. With the habitat-informed calibration, we obtained higher predictive accuracy in cellularity and volume metrics than the local calibration at V3 from just four parameters and a dramatically reduced computational time (median time to calibrate from 153 to 9.1 min). This represents an important step forward in the practical utility of our biology-based modeling approach.

Xu et al.^26^. generated habitats from DW-MRI data acquired before breast cancer patients (any subtype) received NAT. The habitats alone and the habitats in combination with conventional MRI features were used as predictors of pCR in a logistic regression analysis, achieving AUC values of 0.72 and 0.76 in the training set (*n* = 100), respectively, and 0.80 and 0.82 in the testing set (*n* = 43), respectively^26^. Kazerouni et al.^25^. also generate habitats from pretreatment TNBC data (*n* = 46), finding that patients who achieved pCR had a significantly a higher fraction of voxels in their high vascularity, high cellularity habitat. These methods offer the benefit of predicting pCR prior to initiating NAT, but lacks the interpretability offered by a biology-based modeling framework. It is possible to combine data-driven methods to obtain the necessary parameters for a biology-based model *prior* to NAT to obtain both the pre-treatment predictive capabilities offered by previous works and interpretability benefits achieved in the present study. Indeed, we have recently contributed such a study that accomplished this for the special case of global model parameters^49^.

The method has clinical utility as it contributes to the development of accurate and efficient mechanism-based models that can be used to not only predict, but also optimize response to treatment, subsequently improving patient outcomes^10–12,50–54^. For example, using a locally calibrated mechanism based model, Wu et al.. identified optimal therapeutic schedules for TNBC patients, yielding a 20.95–24.76% improvement in pCR rate^12^. Through using our habitat-informed calibration rather than a local calibration, their results could benefit from faster, more accurate predictions of patient-specific response to treatment. This would allow treating teams to efficiently test (i.e., simulate) a suite of therapy schedules to identify the optimal treatment to improve patient response^10,51^. Optimizing response to improve outcomes is of particular importance for TNBC, as approximately half of patient do not attain a pCR to neoadjuvant chemotherapy^4^.

There are multiple opportunities for further investigation. First, the DCE-MRI data used to form the habitats has a higher temporal resolution than what is typically collected in the standard-of-care clinical setting. We rely on the high temporal resolution to calculate the MSI and PEI metrics, so these metrics may require adjustment before applying our method to other data sets. The MSI and PEI could be calculated with lower temporal resolution data, but these maps’ ability to inform habitats would need to be assessed. Alternatively, the SER is available in the clinical setting, thus the ADC + SER habitats could be used to inform the mathematical model in the majority of cases. Second, as we form habitats using longitudinal data, we are assuming that the inter-visit registration pipeline^36^ is quite accurate. While this pipeline has previously been used^8^ and is being further validated on the I-SPY2 dataset^9,55^, future work could benefit from quantifying the effect of the registration on the formation of longitudinal habitats. Third, we do not have histological data available to confirm the biological meaning of our longitudinal habitats, as is done (for example) by Syed et al.^22^. and Jardim-Perassi et al.^24^.. Thus, it would be beneficial to perform longitudinal habitat analysis on a dataset with histological data in the future. Fourth, while the habitat calibration is substantially more efficient than a local calibration through reducing the number of parameters required for calibration, it does not decrease the time of the forward run itself. Thus, to increase efficiency even more, one could employ reduced order modeling to directly increase the efficiency of the forward runs of the model^19^. Finally, we note that the exclusion of patients who did not receive Taxol from all ROC curve analysis inflates the proportion of patients who attain pCR in this portion of our analysis, which may introduce bias in evaluating our ability to predict pCR status.

## Conclusion

We have shown that habitats formed from cell density and perfusion metrics based on MRI data can be used to identify discrete tumor regions for local parameter calibration in a biology-based mathematical model to predict the response of breast cancer to neoadjuvant therapy. The habitats allow for number of calibrated parameters to be reduced by a factor of 162, which leads to a 17× reduction in time to calibrate and a significant increase in predictive accuracy when compared to a voxel-level parameter calibration. When evaluating the calibration fit, the habitat-informed calibration had significantly higher accuracy than the global calibration, but significantly lower accuracy than a local calibration. However, when predicting a future timepoint, the habitat-informed calibration offered a significant improvement in prediction accuracy over a local calibration. Furthermore, there were no deleterious effects on ability to predict pCR at the completion of NAT when compared to the model that employed voxel level calibrations. Therefore, using a small number of habitats to identify which regions require a local parameter value in a biology-based model allows for efficient, interpretable, and accurate predictions of patient response to neoadjuvant therapy.

## Supplementary Information

Below is the link to the electronic supplementary material.

Supplementary Material 1

Supplementary Material 2

Supplementary Material 3

Supplementary Material 4

Supplementary Material 5

## Data Availability

The datasets generated during and/or analysed during the current study are not publicly available due to language in the informed consent document limiting public release of patient level data but are available from the corresponding author on reasonable request.
