# Supplementary material for: Predicting the response of triple negative breast cancer to neoadjuvant systemic therapy via biology-based modeling and habitat analysis
Source: Sci Rep. 2025 Nov 26;15:42111. doi: 10.1038/s41598-025-25989-z (PMC12657976; doi:10.1038/s41598-025-25989-z)
Supplement: Supplementary file 5 — Supplementary Material 5 [file 41598_2025_25989_MOESM5_ESM.docx]

**Supplementary materials: Predicting the response of triple negative breast cancer to neoadjuvant systemic therapy *via* biology-based modeling and habitat analysis**

Casey E. Stowers^1^, Chengyue Wu^1,3,5,9,10^, Clinton Yam^8^, Jingfei Ma^3^, Gaiane M. Rauch^4^, Thomas E. Yankeelov^1,2,3,6,7^*

^1^Oden Institute for Computational Engineering and Sciences

^2^Livestrong Cancer Institutes

Departments of ^6^Biomedical Engineering, ^7^Diagnostic Medicine,

The University of Texas at Austin

Austin, Texas

Departments of ^3^Imaging Physics, ^4^Abdominal Imaging, ^8^Breast Medical Oncology, ^9^Biostatistics

^10^Institute for Data Science in Oncology

The University of Texas MD Anderson Cancer Center

Houston, TX

*Corresponding author

Target journal: Scientific Reports

Please address correspondence to:

Thomas Yankeelov, Ph.D.

Department of Biomedical Engineering

The University of Texas at Austin

107 W Dean Keeton Street Stop C0800

Austin, TX 78712

(512) 471-3604

[tey@utexas.edu](mailto:tey@utexas.edu)

**S1: Supplementary methods**

*S1.1: Patient data*

In Table S1, we present summary information for the patient cohort, including age, therapy received after A/C, histologic type, T category, and N category.

*S1.2: Spatial interaction matrices*

As the habitats were not formed using any spatial information, we computed “spatial interaction matrices” (SIM) using a method developed by Wu *et al*.^1^ to assess spatial connectivity (i.e., how contiguous each habitat is) within the habitats. At each habitat number *H*, we initialize an *H*×*H* SIM for each patient. We visualize how this matrix is populated for a simplified, two dimensional case in Figure S1. For each voxel in the tumor ROI, we consider its surrounding 3×3 set of voxels. In the example in Figure S1, the voxel at the center of the box is part of the yellow habitat, meaning we will add values to the yellow row of the SIM. Zero of the surrounding voxels are part of the blue habitat, so we add zero to the blue column of the SIM. Six of the surrounding voxels are also part of the yellow habitat, so we add six to the yellow column of the SIM. Finally, two of the surrounding voxels are part of the red habitat, so we add two to the red column of the SIM. This process is repeated, adding to the same matrix, for all voxels in the tumor. The same process is followed in the three dimensional case, but we consider the 3×3×3 cube surrounding a voxel, rather than the 3×3 box visualized. Following this process, if most of the voxels in the surrounding cube are in the same habitat as the central voxel of interest, we will add the largest number to the diagonal of the matrix. Thus, large values on the diagonal of the SIM indicate spatial connectivity.

We note that for voxels on the edge of the tumor, we do not add to any column of the matrix for neighbors that are not within the tumor ROI and thus are not assigned a habitat. While forming the matrix, we sum the total number of “interactions” considered (i.e., 26 interactions for a voxel not on the tumor edge, or fewer for a voxel at the edge that has some neighbors outside the tumor and therefore not assigned habitats). We all entries in the matrix by the total number of interactions such that the entries have a maximum value of one. This ensures that tumor size does not impact our analysis of spatial connectivity of the habitats.

To assess the spatial interaction of our habitats, we calculated and normalized the SIM for each patient at each habitat number *h*. We then randomly shuffled the habitat maps for each patient to calculate and normalize another SIM for each habitat number *h*. Across the patient cohort, we aggregated the diagonal values of the habitat SIMs and the randomly shuffled habitat SIMs to provide two distributions. The medians of these distributions were compared using the two-sided Wilcoxon rank sum test with a 5% significance level to determine if the habitat SIMs have significantly higher diagonal values than the randomly shuffled habitat SIMs. This test was performed as significantly higher values on the diagonal indicate more spatial connectivity in our habitats formed *via* a clustering algorithm than our habitats formed *via* random shuffling.

*S1.3: Using habitats as predictors of pCR status*

After the habitats were formed, we compute the percentage of tumor volume in each habitat for each patient. We use these percentages as predictors of pCR status in a receiver operating characteristic (ROC) curve analysis. We then identify the habitat number and individual habitat that provided the highest AUC value for each of the three data options used to form habitats (i.e., ADC+MSI, ADC+PEI, and ADC+SER).

*S1.4: Biology-based model*

In Table S2, we define parameter values and calibration ranges for the biology-based mathematical model presented in Eqs. (7 - 11).

**S2: Supplementary results and discussion**

*S2.1: Habitat formation*

Figures S2 and S3 show the center slice maps for the 2-16 habitats generated for an example patient using the ADC+PEI and ADC+SER data, respectively. As with the analogous Figure 2 for ADC+MSI in the main text, the habitats visually appear to have a degree of spatial connectivity.

To assess the spatial connectivity, we formed a distribution of the diagonal values of the habitat SIMs across the cohort and a distribution of the diagonal values of the randomly shuffled habitat SIMs across the cohort. For each of the ADC+MSI, ADC+PEI, and ADC+SER habitats at all habitat numbers from 2 to 16, we report the first, second, and third quartile for each of these distributions, as well as the p-value from the Wilcoxon rank sum test comparing the distribution medians, in the supplemental excel file “p_values_and_medians_SIMS.xlsx”. In all cases, we found that the median of the distribution of the diagonal values of the habitat SIMs was significantly higher than the median of the distribution of diagonal values of the randomly shuffled habitat SIMs, indicating that the habitats have significantly more spatial connectivity than randomly shuffled habitats.

*S2.2: Habitats as predictors of pCR status*

After forming the habitats, we quantified the ability of the percentage of tumor volume in each habitat to be used as a predictor of pCR in a ROC curve analysis. We tested all three data types for forming habitats (i.e., ADC+MSI, ADC+PEI, and ADC+SER) and all habitat numbers from 2-16. We visualize the ROC curves for each of these three analyses in Figure S4. For the ADC+MSI habitats, we attain the highest AUC at five habitats, giving a mean (95% CI) of 0.78 (0.68 - 0.86), as visualized in Figure S4A. This habitat has a mean ADC at V1 and V2 of 1.1 × 10^-3^ mm^2^/s and 1.3 × 10^-3^ mm^2^/s and a mean MSI 0.23 s^-1^ at both V1 and V2. For ADC+PEI habitats, we attain the highest AUC at 14 habitats, giving a mean (95% CI) of 0.64 (0.51 - 0.75) ), as visualized in Figure S4B. This habitat has a mean ADC at V1 and V2 of 1.0 × 10^-3^ mm^2^/s and 1.2 × 10^-3^ mm^2^/s and a mean PEI at V1 and V2 of 239 s and 242 s. For the ADC+SER habitats, we attain the highest AUC at five habitats, giving a mean (95% CI) of 0.71 (0.60 - 0.80) ), as visualized in Figure S4C. This habitat has a mean ADC at V1 and V2 of 1.1 × 10^-3^mm^2^/s and 1.2 × 10^-3^ mm^2^/s and mean SER at V1 and V2 of 1.0 for both V1 and V2.

Comparing this to previous work, Kazerouni *et al*.^2^ formed habitats from DW- and DCE-MRI data in a cohort of 46 women with TNBC and found that patients who attain pCR have significantly higher fractions of the “high vascularity-high cellularity” habitat at baseline. When considering the best ADC+MSI habitat we identified for predicting pCR by percent volume, we identify a similar trend. The mean ADC at V1 and V2 indicates high cellularity when compared to the overall mean ADC. Additionally, the mean MSI for this habitat is larger than the mean MSI across all voxels, indicating this habitat represents a well vascularized region. Thus, we conclude that the habitat we identified as the best direct predictor of pCR agrees with previous work using habitats to predict pCR. However, it is important to note that while the percent volume in the habitat has similar predictive accuracy to the biology-based models, it lacks interpretability on response dynamics. The biology-based models not only provide similar AUC values for predicting pCR, but also describe patient-specific, spatio-temporal tumor development and offer possibilities for therapy optimization^3,4^.

*S2.2: Selecting optimal habitat number*

We report all *p*-values for comparisons made to select the optimal habitat number to inform the calibrations in the supplemental excel file “p_values_selection_of_optimal_h.xlsx”.

*S2.3: Comparison of global, habitat, and local calibration accuracy*

We report all *p*-values for comparisons made between the global, three habitat-informed, and local calibrations and predictions in the supplemental excel file “p_values_global_habitat_local.xlsx”.

*S2.4: Predicting pCR*

We report all *p*-values for the comparisons of AUC values between the calibration options in the supplemental excel file “p_values_delong.xlsx”.

We provide visualizations of the ROC curves for the ADC+PEI and ADC+SER habitat informed calibrations in Figures S5 and S6.

*S2.5: Calibrated parameter values per habitat*

To ensure that the parameters calibrated to each habitat align with the local parameter calibrations, we show parameter distributions in Figure S7. Figure S7A presents the distribution of the habitat-informed proliferation rate values for each of the three ADC+MSI habitats. The median (IQR) proliferation rate values are 0.051 (0.032-0.079) day^-1^, 10^-6^ (10^-6^-0.0025) day^-1^, and 10^-6^ (10^-6^-0.0036) day^-1^ for habitats one, two, and three, respectively. Figure S7B shows the distribution of the local proliferation rate values for the voxels in each of the three ADC+MSI habitats. The median (IQR) proliferation rate values are 0.055 (0.0031-0.093) day^-1^, 10^-6^ (10^-6^-0.027) day^-1^, and 10^-6^ (10^-6^-0.016) day^-1^ for habitats one, two, and three, respectively. Thus, for both the habitat-informed and local calibrations, habitat one calibrates to higher values than habitats two or three, showing consistency between our calibration methods.

**Supplementary tables**

**Table S1**: Patient information.

| **Characteristics** | **Patient population** | | |
| --- | --- | --- | --- |
|  | Total  *n* = 138 | Non-pCR  *n* = 71 | pCR  *n* = 67 |
| Age, median (range), years | 49 (28-78) | 49 (31-77) | 49 (28-78) |
| Second therapy course | | | |
| Taxol | 101 | 43 | 58 |
| Other | 37 | 28 | 9 |
| Histologic type | | | |
| Invasive ductal carcinoma | 123 | 59 | 64 |
| Metaplastic | 10 | 8 | 2 |
| Invasive mammary carcinoma | 3 | 2 | 1 |
| Poorly differentiated carcinoma | 1 | 1 | 0 |
| Apocrine | 1 | 1 | 0 |
| T category | | | |
| T1 | 25 | 11 | 14 |
| T2 | 96 | 51 | 45 |
| T3 | 14 | 9 | 5 |
| T4 | 2 | 0 | 2 |
| Unstaged | 1 | 0 | 1 |
| N category at diagnosis | | | |
| N0 | 96 | 45 | 51 |
| N1 | 23 | 15 | 8 |
| N2 | 5 | 4 | 1 |
| N3 | 14 | 7 | 7 |

**Table S2**: Model parameters values and calibration ranges

| **Parameter** | **Description** | **Value/calibration range** |
| --- | --- | --- |
| $k(\boldsymbol{x})$ | Proliferation rate at each position $\boldsymbol{x}$ | Calibrated, [10^-6^,0.1] day^-1^.^5^ |
| $\theta$ | Carrying capacity of a single voxel | Fixed, number of spherical tumor cells with radius 10 μm that fit in a voxel with packing density 0.7405.^6^ |
| $\alpha$ | Drug efficacy | Calibrated, [10^-6^,0.8] day^-1^.^5^ |
| $\beta_{j}$ | Decay rate of drug *j* | Fixed, 0.25 and 2.0 days^-1^ for Adriamycin and Cytoxan, respectively.^7–9^ |
| $D_{0}$ | Diffusion coefficient in absence of external force | Fixed, 10^-3^ mm^2^day^-1^.^10^ |
| $\gamma$ | Empirical coupling constant for von Mises stress | Fixed, 2.5 1/kPa.^10^ |
| $E(\boldsymbol{x})$ | Young’s modulus at position $\boldsymbol{x}$ | Fixed, 2, 4, and 20 kPa for adipose, fibroglandular, and tissue, respectively.^11^ |
| $\nu$ | Poisson’s ratio | Fixed, 0.45 (unitless) |
| $\kappa$ | Tumor cell force coupling constant | Fixed, 1 kPa.^12^ |

**Supplementary figures**

**Figure S1**: For an example ADC+MSI habitat map with three habitats, we show how values are added to a spatial interaction matrix (SIM) in a simplified, two dimensional case. For each voxel, we consider the surrounding 3×3 box. In the example, the voxel at the center is part of the yellow habitat. Thus, we set the row of the SIM to the yellow row. Zero of the surrounding voxels are part of the blue habitat, so we add zero to the blue column. Six of the surrounding voxels are part of the yellow habitat, so we add six to the yellow column. Two of the surrounding voxels are part of the red habitat, so we add two to the red column. This is repeated for all voxels in the tumor to provide a final, 3×3 SIM. To extend to the three dimensional case, we consider the 26 neighboring voxels in the surrounding 3×3×3 cube. If the majority of the voxels surrounding the central voxel are in the same habitat, we will add the biggest number to the diagonal of the matrix (as happens in this example). Thus, the diagonal of the SIM quantifies the degree of spatial connectivity, or how spatially contiguous each habitat is, within each habitat.


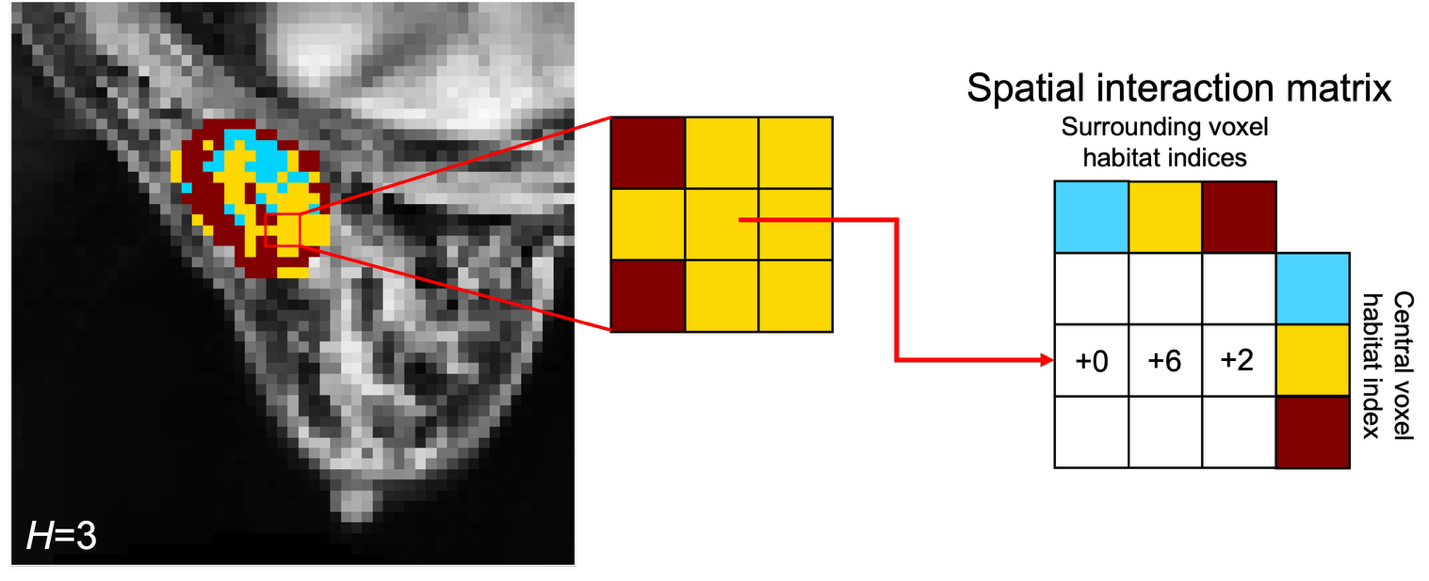


**Figure S2**: The *H* = 2 to *H* = 16 habitats computed using the apparent diffusion coefficient (ADC) and positive enhancement integral (PEI) at V1 and V2 are visualized on the center axial tumor slice of an example patient. As the indices assigned by *k*-means clustering are random, we order the indices in terms of increasing mean V1 ADC to more easily visualize changes in the maps as we increase *H*. While the habitats are not formed using any spatial information, note that they do seem to be spatially connected (see Figure S1). As we increase *H*, the resulting habitats capture increasing intra-tumoral heterogeneity. For example, when *H* = 1, 2, or 3, we identify a small habitat covering a portion of voxels at the bottom of the tumor. This habitat is then divided further into smaller habitats when *H* is larger to capture additional heterogeneity. Since the habitats were used to inform regions in which the tumor cell proliferation rate was locally calibrated, this can inform heterogeneity in the calibrated proliferation rates.


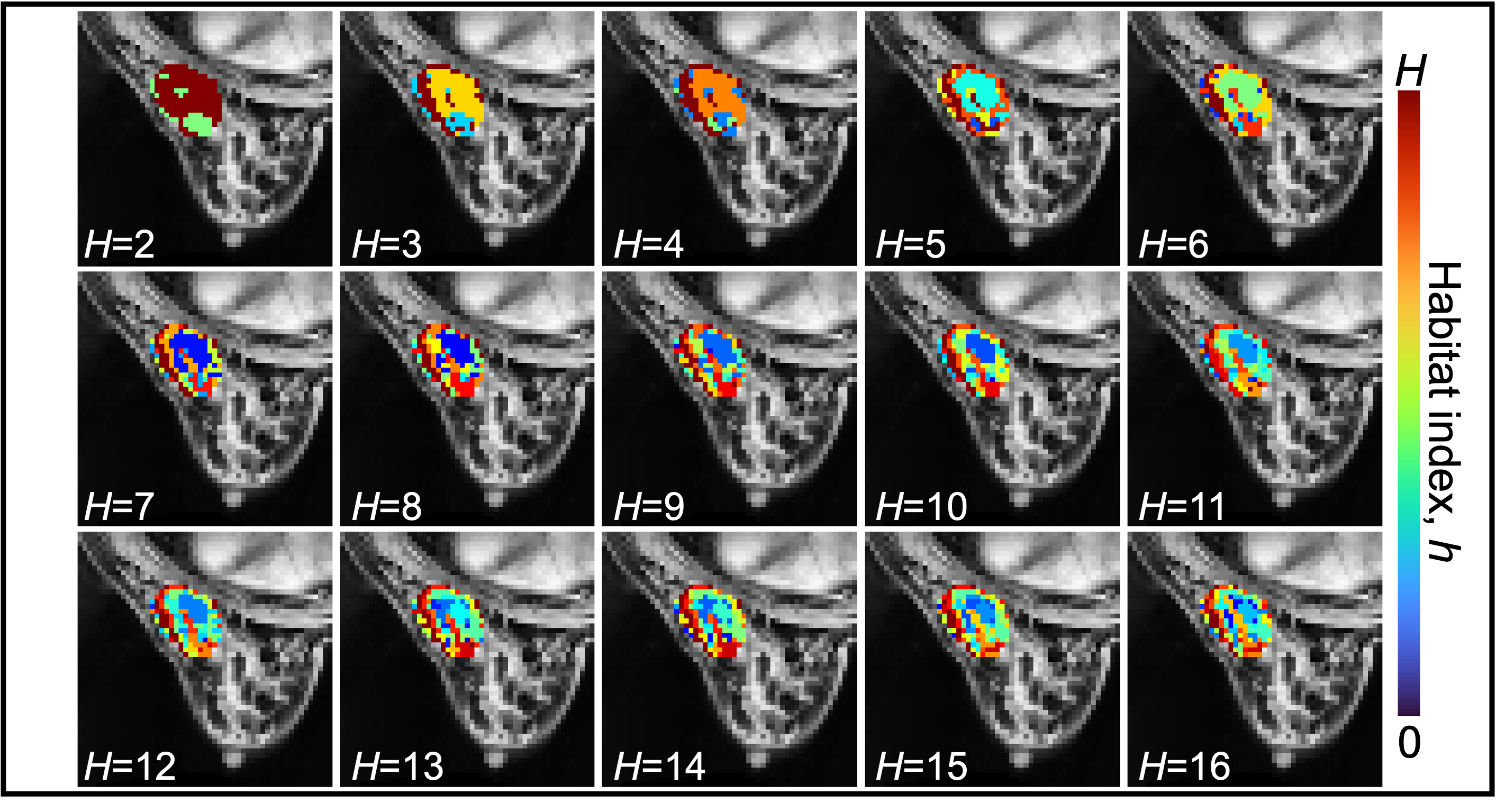


**Figure S3**: The *H* = 2 to *H* = 16 habitats computed using the apparent diffusion coefficient (ADC) and signal enhancement ratio (SER) at V1 and V2 are visualized on the center axial tumor slice of an example patient. As the indices assigned by *k*-means clustering are random, we order the indices in terms of increasing mean V1 ADC to more easily visualize changes in the maps as we increase *H*. While the habitats are not formed using any spatial information, note that they do seem to be spatially connected (see Figure S1). As we increase *h*, the resulting habitats capture increasing intra-tumoral heterogeneity. For example, when *H* = 1, 2, or 3 we identify a habitat covering much of the edge of the left side of this slice of the tumor. A similar region was identified with the ADC+MSI habitats in Figure 2. We again observe that increasing *H* subdivides the habitats from the smaller values of *h*, thereby capturing additional heterogeneity in the calibrated proliferation rates.


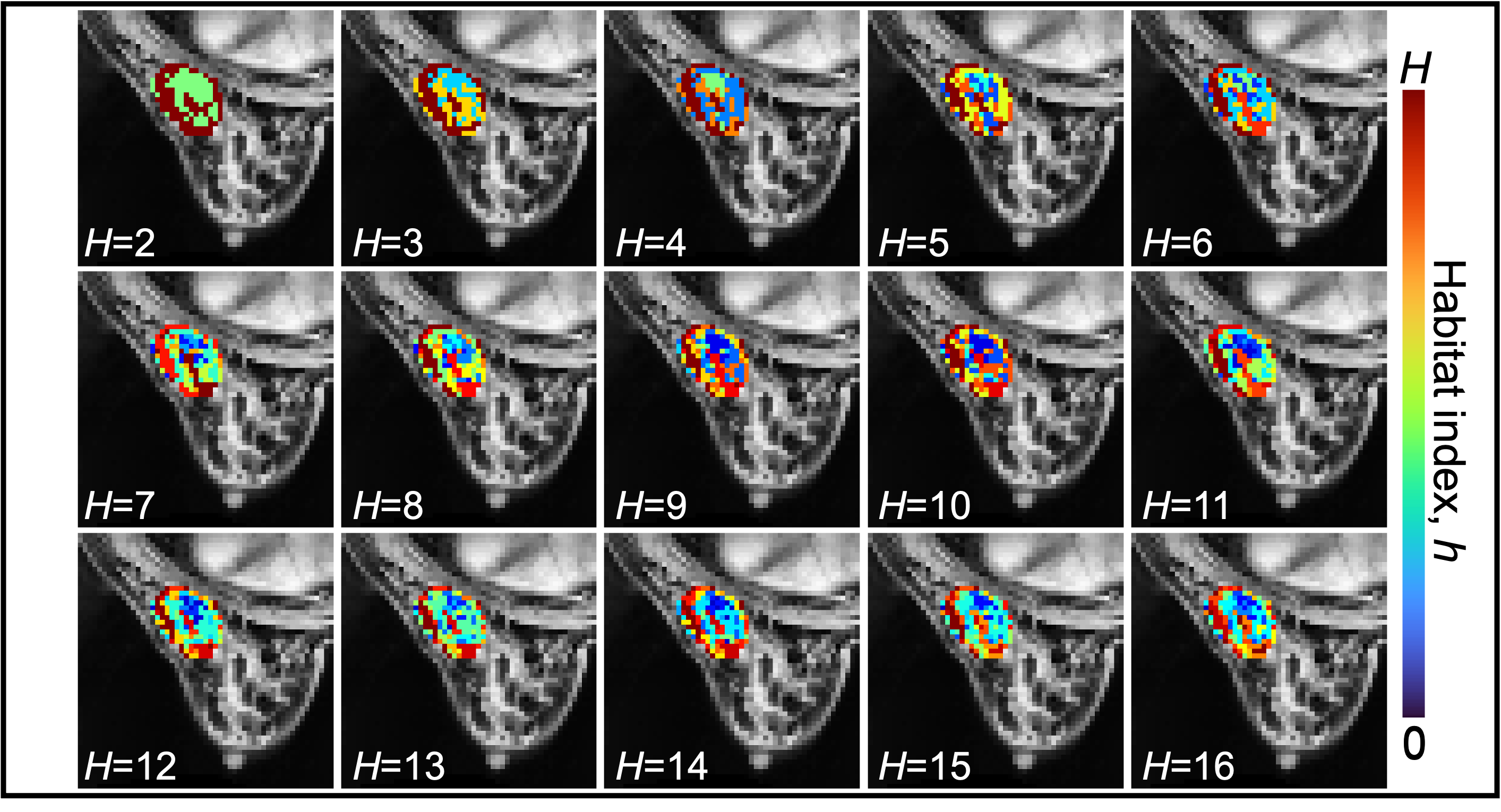


**Figure S4**: Panel **A** shows the ROC curve for predicting pCR status using the percentage of tumor volume in a habitat for the ADC+MSI habitats for *H* = 5. The star denotes the optimal cut point. We obtain an AUC (95% CI) of 0.78 (0.68-0.86). Panel **B** shows the ROC curve for predicting pCR status using the percentage of tumor volume in a habitat for the ADC+PEI habitats for *H* = 14. The star denotes the optimal cut point. We obtain an AUC (95% CI) of 0.64 (0.51-0.75). Panel **C** shows the ROC curve for predicting pCR status using the percentage of tumor volume in a habitat for the ADC+SER habitats for *H* = 5. The star denotes the optimal cut point. We obtain an AUC (95% CI) of 0.71 (0.60-0.80). These results indicate that the ADC+MSI habitats (i.e., panel A) provide the highest accuracy for direct prediction of pCR from the percentage volume within a habitat.

**
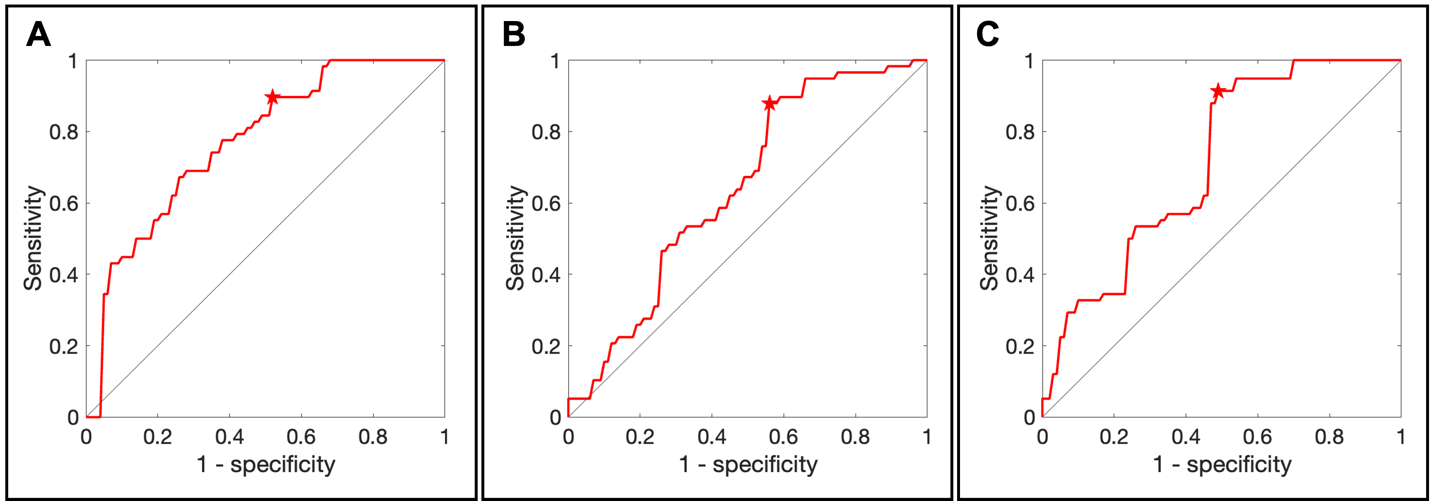
**

**Figure S5**: This figure is identical to Figure 5 in the main text, but with the ADC+MSI habitat-informed calibration replaced with the ADC+PEI habitat-informed calibration. Panel **A** shows the ROC curves for predicting pCR status using V3 total tumor cellularity (TTC). For the ADC+PEI habitat-informed calibration, we obtained AUC (95% CI) values of 0.78 (0.66-0.86). Panel **B** shows the ROC curves for predicting pCR status using V3 total tumor volume (TTV). For the ADC+PEI habitat-informed calibration, we obtained AUC (95% CI) values 0.78 (0.67-0.87). We do not find that the AUC values from any of our calibration options are significantly lower than the AUC from the measured data. Thus, we are able to predict pCR using only V1 and V2 data in an accurate and interpretable fashion without significantly reducing the AUC from pCR predictions using the measured data at V3.


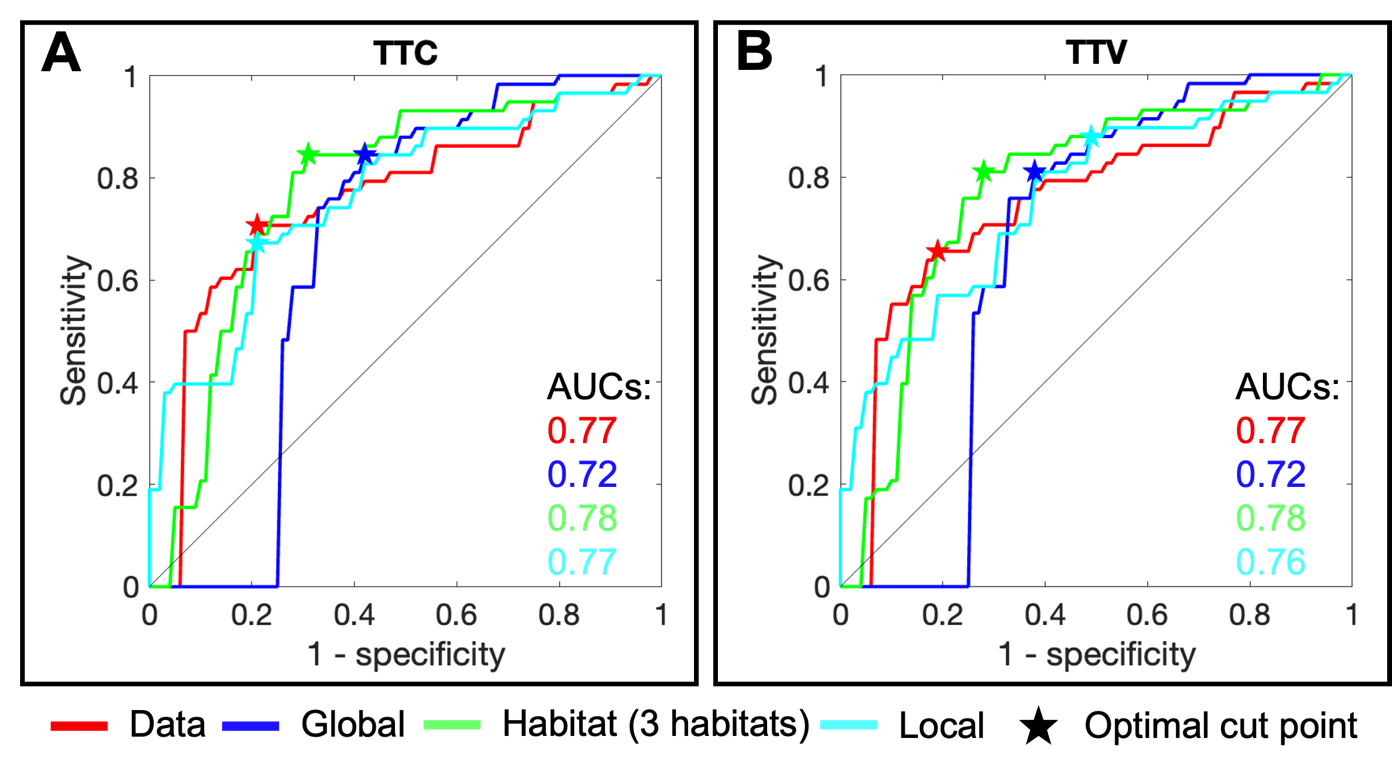


**Figure S6**: This figure is identical to Figure 5 in the main text, but with the ADC+MSI habitat-informed calibration replaced with the ADC+SER habitat-informed calibration. Panel **A** shows the ROC curves for predicting pCR status using V3 total tumor cellularity (TTC). For the ADC+PEI habitat-informed calibration, we obtained AUC (95% CI) values of 0.77 (0.67-0.85). Panel **B** shows the ROC curves for predicting pCR status using V3 total tumor volume (TTV). For the ADC+SER habitat-informed calibration, we obtained AUC (95% CI) 0.78 (0.66-0.86). We do not find that the AUC values from any of our calibration options are significantly lower than the AUC from the measured data. Thus, we are able to predict pCR using only V1 and V2 data in an accurate and interpretable fashion without significantly reducing the AUC from pCR predictions using the measured data at V3.


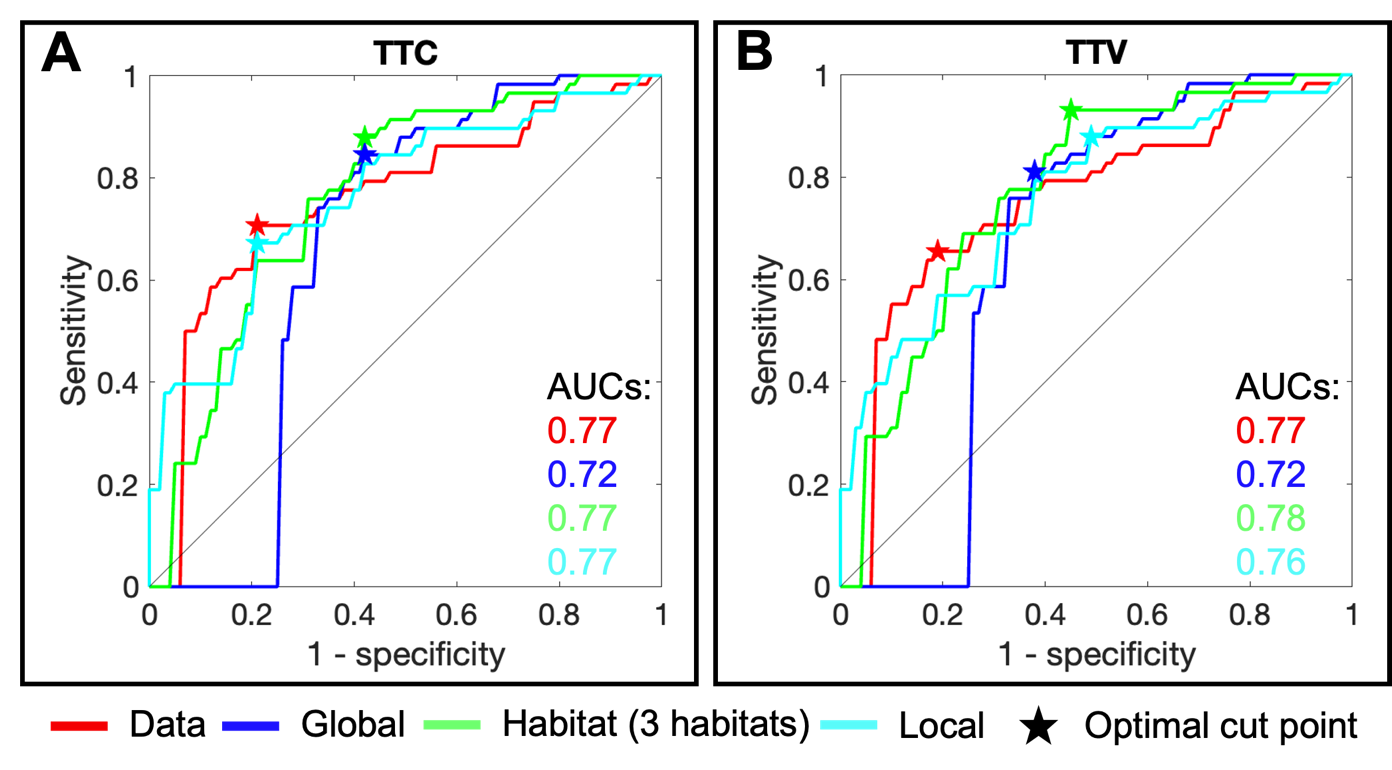


**Figure S7**: In Panel **A**, we plot the distribution of calibrated *k* values across the patient cohort for each of the habitats formed using ADC and MSI data. We observe that habitat 1 has a higher calibrated proliferation rate than both habitats 2 and 3. In Panel **B**, we plot the distribution of the locally calibrated *k* values (i.e., the *k* values calibrated on a voxel-by-voxel basis) across the patient cohort for voxels in each of the habitats formed using ADC and MSI data. This plot shows consistency between our calibration methods, as the voxel-by-voxel local calibration also shows higher *k* values for voxels falling in habitat 1 than in habitats 2 or 3.


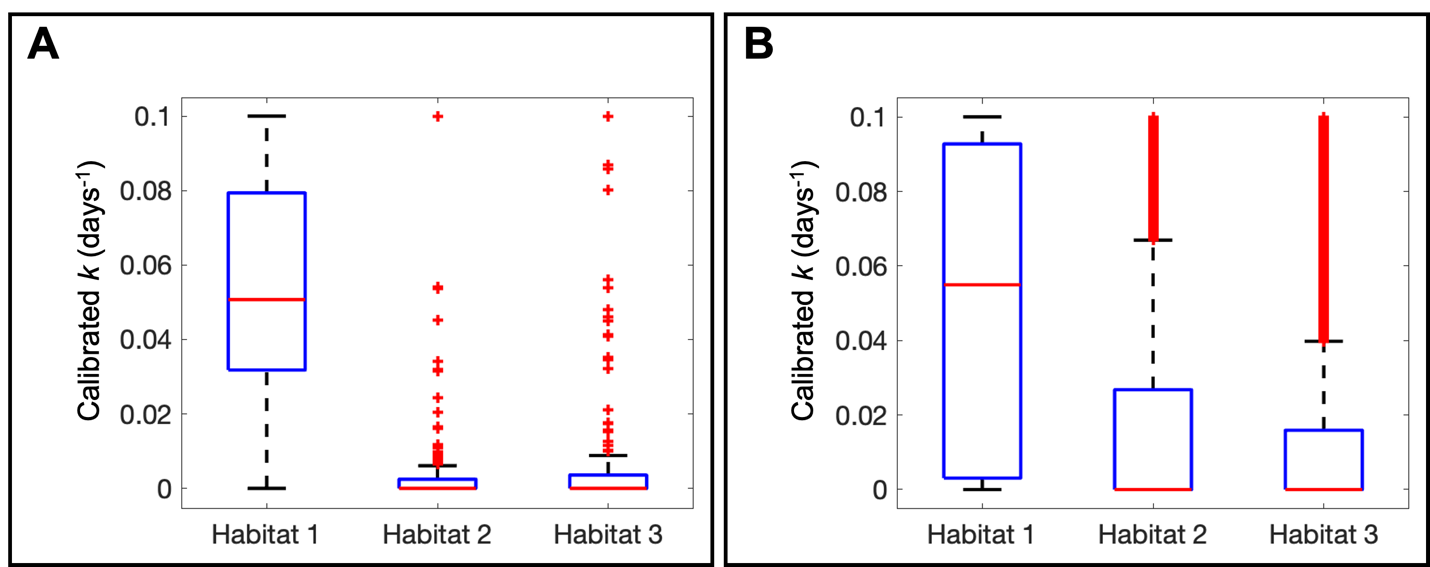


**Supplementary references**

1. Wu, J. *et al.* Intratumoral Spatial Heterogeneity at Perfusion MR Imaging Predicts Recurrence-free Survival in Locally Advanced Breast Cancer Treated with Neoadjuvant Chemotherapy. *Radiology* **288**, 26–35 (2018).

2. Kazerouni, A. S. *et al.* Abstract 5980: Identification of pre-treatment tumor habitats for the prediction of neoadjuvant therapy response in triple negative breast cancer. *Cancer Research* **82**, 5980 (2022).

3. Jarrett, A. M. *et al.* Optimal Control Theory for Personalized Therapeutic Regimens in Oncology: Background, History, Challenges, and Opportunities. *JCM* **9**, 1314 (2020).

4. Jarrett, A. M. *et al.* Evaluating patient-specific neoadjuvant regimens for breast cancer via a mathematical model constrained by quantitative magnetic resonance imaging data. *Neoplasia* **22**, 820–830 (2020).

5. Wu, C. *et al.* MRI-Based Digital Models Forecast Patient-Specific Treatment Responses to Neoadjuvant Chemotherapy in Triple-Negative Breast Cancer. *Cancer Research* **82**, 3394–3404 (2022).

6. Martin, I., Dozin, B., Quarto, R., Cancedda, R. & Beltrame, F. Computer-based technique for cell aggregation analysis and cell aggregation in in vitro chondrogenesis. *Cytometry* **28**, 141–146 (1997).

7. Barpe, D. R., Rosa, D. D. & Froehlich, P. E. Pharmacokinetic evaluation of doxorubicin plasma levels in normal and overweight patients with breast cancer and simulation of dose adjustment by different indexes of body mass. *Eur J Pharm Sci* **41**, 458–463 (2010).

8. Yang, L. *et al.* Pharmacokinetics and safety of cyclophosphamide and docetaxel in a hemodialysis patient with early stage breast cancer: a case report. *BMC Cancer* **15**, 917 (2015).

9. Powis, G., Reece, P., Ahmann, D. L. & Ingle, J. N. Effect of body weight on the pharmacokinetics of cyclophosphamide in breast cancer patients. *Cancer Chemother. Pharmacol.* **20**, 219–222 (1987).

10. Lorenzo, G. *et al.* A global sensitivity analysis of a mechanistic model of neoadjuvant chemotherapy for triple negative breast cancer constrained by in vitro and in vivo imaging data. *Engineering with Computers* **40**, 1469–1499 (2024).

11. Jarrett, A. M. *et al.* Quantitative magnetic resonance imaging and tumor forecasting of breast cancer patients in the community setting. *Nat Protoc* **16**, 5309–5338 (2021).

12. Hormuth, D. A., Eldridge, S. L., Weis, J. A., Miga, M. I. & Yankeelov, T. E. Mechanically Coupled Reaction-Diffusion Model to Predict Glioma Growth: Methodological Details. in *Cancer Systems Biology* (ed. von Stechow, L.) vol. 1711 225–241 (Springer New York, New York, NY, 2018).
